# Placing Ion Channels into a Signaling Network of T Cells: From Maturing Thymocytes to Healthy T Lymphocytes or Leukemic T Lymphoblasts

**DOI:** 10.1155/2015/750203

**Published:** 2015-03-19

**Authors:** Oxana Dobrovinskaya, Iván Delgado-Enciso, Laura Johanna Quintero-Castro, Carlos Best-Aguilera, Rocío Monserrat Rojas-Sotelo, Igor Pottosin

**Affiliations:** ^1^Center for Biomedical Research, University of Colima, 28045 Colima, COL, Mexico; ^2^School of Medicine, University of Colima, 28045 Colima, COL, Mexico; ^3^Departamento de Hematología, Hospital General de Occidente, 45170 Guadalajara, JAL, Mexico

## Abstract

T leukemogenesis is a multistep process, where the genetic errors during T cell maturation cause the healthy progenitor to convert into the leukemic precursor that lost its ability to differentiate but possesses high potential for proliferation, self-renewal, and migration. A new misdirecting “leukemogenic” signaling network appears, composed by three types of participants which are encoded by (1) genes implicated in determined stages of T cell development but deregulated by translocations or mutations, (2) genes which normally do not participate in T cell development but are upregulated, and (3) nondifferentially expressed genes which become highly interconnected with genes expressed differentially. It appears that each of three groups may contain genes coding ion channels. In T cells, ion channels are implicated in regulation of cell cycle progression, differentiation, activation, migration, and cell death. In the present review we are going to reveal a relationship between different genetic defects, which drive the T cell neoplasias, with calcium signaling and ion channels. We suggest that changes in regulation of various ion channels in different types of the T leukemias may provide the intracellular ion microenvironment favorable to maintain self-renewal capacity, arrest differentiation, induce proliferation, and enhance motility.

## 1. Introduction

T cell acute lymphoblastic leukemias (T-ALL) are aggressive neoplastic disorders of the lymphoblasts committed to the T lineage. T-ALL accounts for 15% of pediatric and 25% of adult ALL cases [1]. It is widely accepted that the T cell leukemogenesis is tightly related to the normal T cell development. Various genetic errors during T cell maturation may cause the healthy progenitor to convert into a leukemic precursor cell that lost its ability to differentiate but possesses high potential for proliferation and self-renewal. Accordingly, leukemogenesis is a multistep process, where the genes encoding proteins implicated in the normal T cell development are deregulated. Among them there are transcriptional factors and tumor suppressors, receptors and signal transduction molecules, secreted molecules and growth factors, ion channels, and transporters. Specific genetic alterations define distinct groups of T-ALL with different profiles and levels of gene expression denominated as a gene expression signature. Moreover, gene expression signatures may vary in every special clinical case. Although numerous experimental and clinical reports and detailed reviews dealing with T-ALL are available, the relationships between various components of transcriptional and signaling regulatory networks are very complex and many issues are still to be addressed.

In the present review we are going to reveal a relationship between different abnormalities that drive the T cell neoplasias, with special accent on those occurring in the expression of ion channels in this type of lymphoproliferative disorders. We suggest that changes in regulation of various ion channels in different types of the T-ALL may provide an intracellular ion microenvironment favorable to maintain self-renewal capacity, arrest differentiation, induce proliferation in T cell precursors, and enhance their motility.

We first review normal T cell maturation and recurrent cytogenetic abnormalities reported in the T-ALL, with their relation to main signaling pathways that contributed to leukemogenesis. Next, we address the question how Ca^2+^ signals may be involved in the T-ALL signaling network. Then we provide an overview of the current knowledge on the abnormal expression of ion channels in leukemias, from the point of view of their possible contribution to shaping and maintenance of Ca^2+^ signal, and other mechanisms where ion channels may be involved. And finally, we will discuss the possibility of targeting ion channels to improve the existing protocols of the T-ALL treatment.

## 2. T Cell Maturation in the Thymus

**Figure 1:**
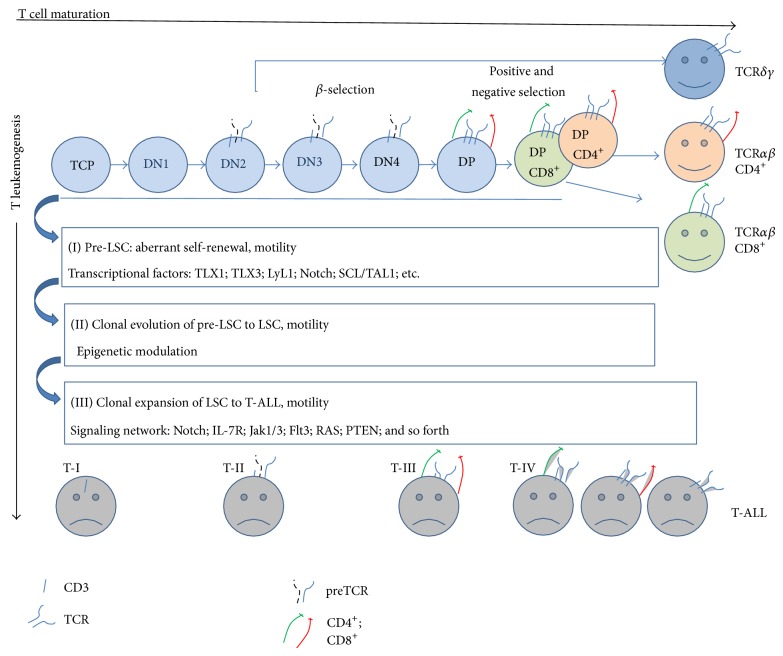
Hierarchical mutagenesis during T cell maturation causes different types of T-ALL (see text for details).

It is widely accepted that T leukemogenesis is a multistep process where several genetic lesions drastically mislead the normal thymocyte maturation [2]. A short overview of key events in early thymocyte development and their links to the leukemogenesis is presented at Figure 1.

T cells can be distinguished from other lymphoid lineages by the presence of the unique antigen-specific T cell receptor (TCR) on the cell surface. TCR is a transmembrane heterodimer composed of two chains, either *αβ* or *γδ*. T cells of TCR*αβ* lineage constitute the bulk of T cell populations in lymphoid organs and recognize antigen-derived peptides bound to the molecules of a major histocompatibility complex, of classes I or II (MHC-I or MHC-II), on the surface of antigen-presenting cells. T cells of TCR*γδ* lineage are generally not MHC-restricted and particularly play an important role in protection of the mucosal tissues from the external infection ([3, 4]; revised in [5, 6]). Intracellular signaling through TCR depends on its association with a multimeric complex of membrane proteins referred to as CD3 and composed of four distinct polypeptide chains that assemble and function as three pairs of dimers (*εγ*, *εδ*, and *ζζ*). Accordingly, TCR/CD3 protein complexes are defining features of T lineage and therefore are used as T cell markers. In addition, mature TCR*αβ* lymphocytes bear CD4 or CD8 transmembrane proteins that serve as coreceptors for TCR in two subpopulations: T helpers (CD4^+^) and cytotoxic T cells (CD8^+^). The extracellular domains of CD4 and CD8 bind to conserved regions of MHC class II and MHC class I molecules, respectively. The coengagement of MHC molecule by both TCR and CD4 or CD8 enhances the avidity of T cell binding to its target and helps to initiate the cascade of intracellular signaling events.

Each of the several millions of T cells circulating in the organism possesses a unique TCR capable of recognizing its own MHC molecules, which present specific antigenic structure, distinct for every T cell clone and without cross-reactivity to self-antigens. Maturation of self-tolerant T cells, which differ in specificity of their TCR receptors and which are restricted to self-MHC, takes place in the thymus. The broad repertoire of TCR is generated by strictly ordered gene rearrangements in TCR loci encoding *α*, *δ*, *β*, and *γ* chains. The genomic locus coding every TCR chain contains gene clusters corresponding to the variable (V), the diversity (D), the join (J), and the constant (C) regions. Functional TCR genes are produced by the recombination process that assembles V, D, J, and C segments dispersed along a large genetic locus into a single transcriptable gene. Recombination activating genes RAG1 and RAG2 and terminal deoxynucleotidyl transferase (TdT) play a central role in the TCR rearrangement. At this phase, only those thymocytes survive, in which genetic rearrangements were productive and resulted in the appearance of a final unique lineal coding sequence of TCR chains. The apoptotic program is triggered in the rest of cells, which managed the rearrangement task poorly [5, 6]. The earliest T cells, lacking detectable CD4 and CD8 (CD4^−^CD8^−^), are, therefore, referred to as double-negative (DN) cells. Later on, they start to express both CD4 and CD8 (CD4^+^CD8^+^) and are denominated as double-positive (DP) cells. Finally, DP differentiates into single positive (SP) cells, either CD4^+^CD8^−^ or CD4^−^CD8^+^, which leave to periphery. DN T cells are subdivided into four subsets (DN1-4), based on the presence or absence of other cell surface molecules, including CD117, the receptor for stem cell growth factor c-kit; CD44, an adhesion molecule; CD25, the *α* chain of the IL-2 receptor (IL-2R), determining the IL-2R affinity [5, 7]. In every DN stage, characteristic events of TCR rearrangements take place. DN1 thymocytes express only c-kit and CD44 (c-kit^++^ CD44^+^ CD25^−^), but once they encounter the thymic environment and become resident in the cortex, they express CD25 and proliferate, becoming DN2 thymocytes (c-kit^++^ CD44^+^ CD25^+^). During this stage, rearrangement and transcription of germ line D*β* and J*β* segments belonging to TCR*γ* and TCR*δ* gene locus begin. However, the TCR*α* locus does not rearrange, because the regions of DNA encoding TCR*α* genes are not yet accessible to the recombinase machinery. At the late DN2 stage, T cell precursors are fully committed to the T cell lineage and reduce expression of both c-kit and CD44. Cells in transition from the DN2 to DN3 (c-kit^+^ CD44^−^ CD25^+^) stages continue rearrangement of the TCR*γ*, TCR*δ*, and TCR*β* chains, start to express CD3, and make the first major decision in T cell development: whether to join the TCR*γδ* or TCR*αβ* lineage [4, 5, 8, 9]. The choice to become a *αβ* or *γδ* T cell is dictated by when and how fast the genes, coding for each of the four receptor chains, successfully rearrange. Rearrangement of the *β*, *γ*, and *δ* loci begins during the DN2 stage. To become a TCR*αβ*, T cell must generate a TCR*β* chain—an event that depends on successful VDJ rearrangement. To become a TCR*γδ*, however, a thymocyte must generate two functional proteins that depend on two separate in-frame rearrangement events [5]. Germline V*β* transcription and rearrangement to assembled DJ*β* complex occur in DN3 cells. Those DN3 cells that successfully rearrange their *β* chain and therefore commit to the TCR*αβ* lineage lose expression of CD25 halt proliferation and enter the final phase of their DN stage of development, DN4 (c-kit^low/−^ CD44^−^ CD25^−^), which maturate directly into DP thymocytes [5, 8]. Cellular differentiation involves epigenetic changes that regulate the transcription of genes encoding lineage-specific proteins and pluripotency factors. Developmental stage-specific regulation of transcriptional accessibility helps control V(D)J or VJ recombination. For example, V*β* segments on nonrearranged TCR*β* alleles are accessible in DN thymocytes, when they recombine, and inaccessible in DP thymocytes, when they do not rearrange [8]. Assembly and expression of functional TCR*γ* and TCR*δ* chains that can pair to form TCR*γδ* complexes in DN2/3 thymocytes drive cellular proliferation and promote differentiation into *γδ*T cells. In contrast, DN thymocytes that have successfully rearranged their TCR*β* chains are valuable and are identified and expanded via a process known as *β*-selection. At this stage of development, DN3 thymocytes express the unique pre-T*α* chain. It acts as a surrogate for the real TCR*α* chain, which has yet to rearrange, and assembles with a successfully rearranged and translated TCR*β* chain, as well as CD3 complex. This precursor TCR/CD3 complex, known as the pre-TCR, is an important player in the next stages of thymocyte maturation. It initiates a signal transduction pathway resulting in maturation to the DN4 stage (c-kit^−^ CD44^−^ CD25^−^), rapid proliferation in the subcapsular cortex, and suppression of further rearrangement of TCR*β* chain genes, resulting in allelic exclusion of the *β*-chain locus, and induces development to the DP stage. After entering into the DP stage, cessation of proliferation and initiation of TCR*α* chain rearrangement occurs. Once a TCR*α* chain has successfully rearranged, it will dimerize with the TCR*β*, replacing the pre-T*α* chain. The mature TCR*αβ* generates signals that lead to the next stages of positive and negative selection. During positive selection, only T cells are able to recognize host MHC survives, ensuing MHC-restriction. T cells that recognize self-MHC molecules and peptides with high affinity are deleted from the repertoire of cells during negative selection, providing self-tolerance [7]. The vast majority of DP thymocytes (more than 95%) never meets the selection criteria and dies by apoptosis within thymus. Upon its positive selection, TCR*αβ* activates intracellular signals that stop TCR*α* rearrangements and promote differentiation of DP cells into SP thymocytes, which leave the thymus as CD4^+^ or CD8^+^ TCR*αβ* cells [4, 5, 7, 8].

Key steps in T cell maturation are controlled by several transcriptional regulators. The most important players in T cell ontogeny that are afterward deregulated in T leukemogenesis are Notch receptors proteins, and transcriptional factors of helix-loop-helix (HLH, the E2A, and HEB) and Homeobox (HOX) families [2, 6, 10].

Notch signaling pathway is evolutionarily conserved and operates in many cell types of different tissues at various developmental stages [11]. It is an important coordinator of different stages of the T cell maturation prior to the DP stage, including self-renewal of common lymphoid progenitor (CLP), commitment decision of the CLP toward T cell versus B-cell fate choice, and assembly of pre-TCR in immature thymocytes [2, 12, 13]. Mammals possess four Notch receptors (Notch 1–4) and five corresponding ligands (Delta-like 1, 3, 4, and Jagged 1 and 2). Mature Notch (1–4) receptor is a heterodimer that consists of Notch extracellular (NEC) and Notch transmembrane (NTM) domains associated noncovalently with the heterodimerization domain (HD). Ligand binding initiates the chain of proteolytic cleavages in NEC, culminating in the formation of intracellular Notch (ICN). Subsequently ICN translocates to the nucleus, to form a part of the large transcription activator complex. Notch signaling is regulated at multiple levels. Primarily, expression of Notch receptors and their ligands is restricted to a certain cell population within certain context. Another level of regulation is to insure that ICN is a short-lived protein, due to ubiquitylation within its degradation PEST domain rich in proline (P), glutamic acid (E), serine (S), and threonine (T) [11].

Notch1 activation in maturing thymocytes occurs upon engagement with its ligand, expressed on the thymic stromal cells [14, 15]. Among multiple Notch target genes, identified in T cells, there are transcriptional factors hes1 (and hes-related genes), Myc, NFAT, and NF*κ*B [13].

Myc exhibits a steady expression at early stages and an abrupt drop in DP thymocytes [16]. A burst of proliferation, triggered by *β*-selection, requires preTCR, Notch, and Myc signaling [17] and is augmented by IL-7 [10]. The preTCR expression shuts down Notch signaling and therefore negatively regulates these mitogenic pathways [10]. Transcriptional factors NFAT and NF*κ*B were shown to be upregulated in thymus at the transition of DN cells to the DP stage governed by pre-TCR signaling [18, 19]. Both NF-*κ*B and NFAT regulate the transcription of genes, encoding cytokines, antiapoptotic proteins, and cell cycle regulators [20], and their activation is related to mitogen-activated protein kinase (Raf-MEK-Erk) pathway during positive selection [21].

E2A and HEB proteins bind DNA at specific E-box sites in the enhancers of many T cell specific regulatory genes like CD4 and preT*α* [2]. Members of the HOX family contribute, in some phases of early development, to coordinating the differentiation block, expression of the IL7 receptor, and choice of the *αβ* versus *γδ* lineage in DN2 [2].

Other important regulators of T cell maturation are numerous cytokines, produced by thymocytes themselves or by thymus stromal cells [22]. Among them, IL-2 and IL-7 are of special importance. IL-2 is an autocrine factor coming into the play as early as at DN2 (see above) and regulating the TCR-dependent clone expansion from this moment over the entire life of a T cell. In contrast, IL-7 is paracrine thymic cytokine produced by stromal cells in subcapsular zone, where DN cells are located. IL-7 participates in a coordination of the basic processes of early thymocyte development, namely, survival (through Bcl-2 upregulation), proliferation, and TCR rearrangement.

## 3. T-ALL as a False Mirror of the T Cell Maturation

As far as neoplastic transformation may occur at different stages of T cell differentiation (Figure 1), T-ALLs represent a very heterogeneous group of tumors with regard to their immunophenotype, cytogenetic, and clinical features and response to treatment. Arrest of differentiation program at specific stage of normal thymocyte development is a priming event in the T leukemogenesis. Simultaneously, uncontrolled cell growth and clonal expansion occur as a result of several mutations in the genes, involved in regulation of cellular metabolism, cell cycle control, and self-renewal of stem cells. The most of T-ALL oncogenes are downregulated at early stages of the thymocyte development or are not at all expressed in the normal thymus. Different mechanisms of genetic structural rearrangements are implicated in the T-ALL leukemogenesis: (1) translocations involving TCR loci, (2) gene fusion encoding chimeric proteins, and (3) deletions of tumor suppressive genes. As a result, corresponding gene is upregulated (activating mutation) or downregulated (suppressing mutation) (for detailed review, see [2]).

A hierarchical model of mutations, which contributed to the T leukemogenesis, was recently proposed [23]. In accordance with this model, the leukemogenesis occurs in several consequent steps (Figure 1). At the first stage, genetic alterations of transcription factors, leading to the activation of self-renewal program, occur in the immature T cell progenitors and generate the preleukemic stem cells (pre-LSCs). Self-renewal phenotype is essential for acquisition and accumulation of subsequent mutations. Activating mutations of signaling pathways important for T cell maturation allow expansion of pre-LSC, independent of the thymic microenvironment (niche). At the next stage, acquisition of mutations in epigenetic regulators results in transformation of pre-LSCs in LSCs. Finally, apparent T-ALL is generated by a clonal expansion of LSCs, retaining activating mutations in the cytokine signaling pathways.

Considering a rearrangement of gene loci, encoding variable regions of the TCR chains as a key event during the T cell maturation, it is not surprising that, in more than 30% of T-ALL patients, oncogenes are activated while being translocated and juxtaposed to one of the TCR loci [24, 25]. Partner oncogenes, involved in the TCR gene translocation, encode developmentally regulated transcription factors and signaling molecules. They are transcribed simultaneously at early stages of the thymocytes maturation and possess open chromatin configuration, which is vulnerable to the action of recombinase enzymes RAG1 and RAG2. As a result, target genes are put adjacent to strong promoter or enhancer elements of the TCR genes.

The data about frequency of cytogenetic and molecular changes in T-ALL clinical cases are reviewed in detail elsewhere [2, 26]. Here we present summarizing remarks of the most frequent genetic lesions. Microarray-based gene expression analysis revealed that T-ALL patients cluster into four major groups based on the aberrant, subtype-specific expression of transcriptional factors TLX1 or TLX3 (HOX family), LYL1 (HLH family), and TAL1 oncogenes [27].

TLX1 and TLX3 are normally involved in the early embryogenesis being implicated in the organogenesis and differentiation of specific cell types. They are not expressed in developing T cells but seem to be involved in spleen development. In leukemogenesis, they are the most frequent aberrantly expressed genes becoming active due to the translocation involving the TCR loci [1, 28]. TLX1 is expressed in 7% of children and in about 30% of adults with T-ALL, displaying an early cortical phenotype. TLX3 is detected in 20% of children and in 13% of adults. Specific mechanisms of T cell transformation downstream of TLX1 involve the repression of the TCR*α* enhanceosome activity, the blockage of TCR*α* rearrangement, and downregulation of mitotic control genes, which induces the loss of the mitotic checkpoint in nontransformed preleukemic thymocytes [29, 30].

The LYL1 gene is not normally expressed in T lineage. Its upregulation in the T-ALL is due to chromosomal translocation, which juxtaposes it with the T cell receptor *β* gene locus. LYL1 reflects an early arrest in the T cell differentiation. Accordingly, related T-ALLs universally express the early hematopoietic marker CD34 and for the most part lack the expression of both CD4 and CD8 [31]. LYL was shown to be also important for the angiogenesis [27]. Overexpression of TLX3 and LYL1 in leukemic patients correlates with a worse prognosis [27].

TAL1 is a homologue of LYL1. It is involved in the embryonic and adult hematopoiesis and in the angiogenesis but is not normally expressed in maturating T cells. It has been shown that TAL1 and its partners LMO1/2 are coexpressed in the most primitive thymocytes [2]. Genetic evidences that the TAL-1 aberrant overexpression may involve t(1;14) (p32:q11) translocation or submicroscopic interstitial 1p32 deletion with resulting fusion of TAL1 with SIL promoter were provided. TAL1/SCL induces leukemia by inhibiting the transcriptional activity of E47/HEB and interfering with several E47/HEB target genes critical for the thymocyte differentiation [32]. TAL1-positive leukemias show the transcriptional upregulation of CD3 and TCR genes, developmental arrest in DP stage, and an overexpression of antiapoptotic gene Bcl2 [1, 33]. Recently, Sanda and coworkers (2012) have identified a set of transcriptional regulators that collaborate with TAL1 to generate a “core” regulatory circuit that contributes to the initiation and maintenance of human T-ALL [34]. Among TAL1 regulatory partners other transcriptional factors like LMO1/2, HEB, E2A, GATA3, and RUNX1 were found among others. An elevated expression of the TAL1/SCL is detected in over 60% of children and adults [35]. Patients with TAL1/SCL activation respond poorly to existing therapy, ensuing that no more than 50% of patients survive 5 years after diagnostics [1].

Cryptic deletion of the INK4/ARF locus is another frequent anomaly, detected in about 65% T-ALL, which results in cell cycle control defects [2].

Notch mutations are very frequent genetic alterations found in over 50% of T-ALL clinical cases, irrespective to their stage of the differentiation arrest [27, 36]. In contrast to T-ALL, Notch1 mutations are not found in B-ALL [36] and are seen only rarely in acute myeloid leukemia (AML) [37]. Since Notch coordinates self-renewal program of early lymphoid progenitors, activating Notch1 mutations increase the self-renewal capacity of the LSC, resulting in their susceptibility to acquire and accumulate additional genetic abnormalities. Thus, Notch1 signaling deregulation is considered to be crucial for the T cell leukemogenesis. Most common causes in Notch1 signaling deregulation are activating mutations clustered in regions coding HD and PEST domains [38], whereas HD mutations seem to enable the ligand-independent Notch cleavage resulting in the constitutive activation of the Notch protein [39], PEST domain mutations are thought to stabilize the structure and prolong the half-life of the active Notch 1 [40]. In rare cases (<1% of T-ALL), the expression of truncated ligand-independent and constitutively active Notch1 receptor is caused by rearrangement translocation, which juxtaposes the C-terminal region of human Notch1 gene to the TCR-*β* enhancer [41]. Additionally, the half-life of Notch protein may be also increased due to loss-of-function mutations in the FBXW7 gene, coding for a component of ubiquitin ligase complex. In addition to the Notch1, it degrades various proteins, important for the T-ALL pathogenesis such as Myc and cyclin E. The FBXW7 mutations result in inability to bind to its target proteins (Notch1) or bind its targets but fail to tag them for degradation (Myc), in both cases prolonging their half-life. FBXW7 gene lesion was identified in various T-ALL cell lines and in significant number (20%) of T-ALL patients, most of those undergoing disease relapse and resistance to treatment [1, 28].

**Figure 2:**
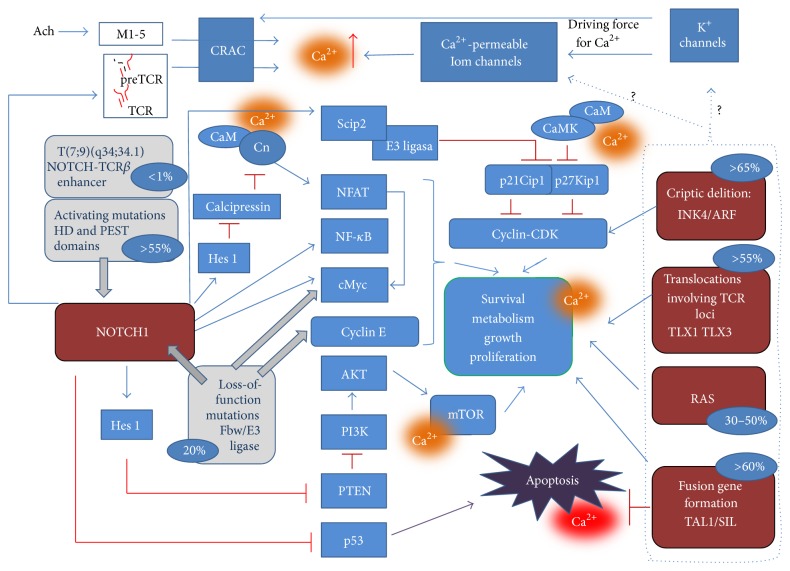
Ca^2+^-dependent signaling pathways in T-ALL. Ca^2+^ influx may occur through CRAC channels, activated in receptor-dependent manner: (a) muscarinic receptors (M1–M5) are stimulated by Ach produced by leukemic cells themselves, but mechanisms of elevated Ach production are not studied yet; (b) TCR or preTCR receptors are activated through mechanisms which may engage Notch upregulation. Another mechanism for Ca^2+^ influx involves activation of nonselective Ca^2+^-permeable channels, activated by different mechanisms. Driving force for sustained Ca^2+^ influx is generated by K^+^ efflux through selective K^+^ channels. Most important genetic lesions and signaling pathways are indicated, together with percentages for each type of lesion recognized in clinical cases. For more details, see the text.

Aberrant Notch1 activation in T-ALL is suggested to promote deregulated proliferation and prevent apoptosis. Molecular mechanism of Notch-mediated cell-cycle progression was shown to involve activation of c-myc [36, 42], NFAT [43, 44], and AKT/PI3K pathway and inhibition of PTEN expression [45] (Figure 2). Among the targets, activated by the Notch1 in the T-ALL, transcriptional factors HES1, HERPI 1&2, and EGF-containing fibulin—like extracellular matrix protein 1 (EFEMP1), vascular endothelial growth factor VEGF, inhibitor of DNA binding 1 (ID1), and SnoRNAs of the box H/ACA quantitative accumulation (SHQ1), immune associated nucleotide 4 like 1 IAN4L1/GIMAP5, and coreceptor CD28 were also reported [46, 47]. Notch1 upregulation enhances the G1/S transition through the induction of Skp2 expression; Skp2 is the component of E-3 ligase complex that degrades p27Kip1 and p21Cip1, inhibitors of cyclin-CDK2/4 complexes [48]. NF-*κ*B cascade seems also to be activated by the Notch1 upregulation, and attenuation of NF-*κ*B resulted in T-ALL suppression, both* in vivo* and* in vitro* [49]. Inhibition of apoptosis may occur through Notch1 activation of different pathways, including the NF-*κ*B or the PKB/AKT/mTOR ones, leading to the p53 inhibition [25].

The question whether Notch aberrant expression is sufficient to induce T-ALL on its own was addressed. For this, different human gain-of-function Notch1 alleles were tested for their ability to drive an ectopic T cell development and to induce leukemia, when expressed in murine bone marrow progenitors [40, 50]. It was shown that the induction of the T cell leukemia is dependent on the Notch1 signal strength. Only rare Notch1 mutations with strong downstream signaling were able to drive the T cell leukemia* per se*, whereas common weak gain-of-function alleles were effective only in combination with a constitutively active Ras oncogene; nonetheless they gave rise to tumors sensible to inhibition of Notch1 signaling. Thus, Notch1 mutations, being indispensable for the majority of clinical T-ALL, require additional mutations in order to drive the leukemogenic process.

Ras proteins play a critical role in the transmission of survival signals from the cell membrane receptors to the intracellular transduction pathways. Mutations of RAS genes are common and have been described in various malignancies including acute leukemias [2]. They lead to the constitutive activation of the RAS-MAPK signaling cascade. Indeed, RAS lesions and activation of the tyrosine kinase genes, as a result of FLT3 and Janus kinase JAK1 mutation, or due to the ABL1/JAK2 gene fusion, have been identified, according to different studies, in 30 to 50% of clinical T-ALL cases [2]. The interest to identify these aberrations resides in the fact that they involve tyrosine kinases, for which specific inhibitors are known. These pathways are attractive candidates for a targeted therapy [51]. The AKT pathway plays a key role in the cell cycle progression and differentiation. Activation of AKT, either through PTEN loss-of-function mutations, activating mutations in the phosphatidyl inositol-3 kinase (PI3K) subunits, or AKT1 itself seem to be relatively common and occurs in nearly half of the (adult) T-ALL cases [52].

Genetic abnormalities that cause leukemia should meet a special favorable microenvironment to be realized. In pathways upregulated in the T-ALL, many functional elements depend directly or indirectly on the Ca^2+^ signaling (Figure 2).

## 4. TCR and Ca^2+^ Signaling in the T Cell Physiology

In resting healthy T cells, the concentration of free intracellular Ca^2+^ ([Ca^2+^]_i_) is kept about 100–200 nm [53]. Changes in concentration of [Ca^2+^]_i_ represent ubiquitous signaling mechanism that regulates different phases of T cell physiology, including the proliferation, lineage decision in the maturation, and antigen activation [53–55]. To be able to maintain low [Ca^2+^]_i_ in resting cell or generate Ca^2+^ signal of a particular frequency and amplitude, time course, and intracellular location, every cell possesses the unique set of components, involved in this regulation, a so-called Ca^2+^ toolkit [56]. Ca^2+^ toolkit includes external sensors (plasma membrane receptors), signal transducers (such as G proteins and phospholipase C isoforms), signal-generating Ca^2+^-permeable ion channels, selective K^+^ channels that hyperpolarize plasma membrane and generate a driving force for Ca^2+^, ER-localized Ca^2+^ storage proteins (such as calreticulin and calsequestrin), signal terminators that serve to return intracellular Ca^2+^ levels to prestimulation levels, such as the ER- and plasma membrane-localized Ca^2+^ pumps (SERCA and PMCA, resp.), plasma membrane exchangers, mitochondria and cytosolic buffer proteins, and Ca^2+^ sensors and effectors such as calmodulin (CaM) and its downstream targets, including CaM kinase (CaMK) and Ca^2+^/calmodulin-dependent serine/threonine phosphatase calcineurin (Cn) and protein kinase C (PKC). The Ca^2+^ toolkit may be modified during every developmental stage according to the changes in signaling events or may be remodeled in tumor cells to sustain the proliferation and avoid the cell death [56].

### 4.1. Mature T Cells

Antigen recognition through TCR receptor is a key event in the T lymphocyte physiology, leading to cell activation, clone expansion, and differentiation to effector cells (reviewed in [57]). Binding of the TCR to the MHC-Ag results in the assembly of the TCR/CD3 (TCR*αβ*/CD3*γεδεζ*2) signaling complex, which is formed by membrane-resident molecules that are physically segregated in resting T cells. The TCR recognition module governs MHC-Ag recognition and the association of the protein tyrosine kinase (PTK) zeta-chain-associated protein kinase of 70 kDa (ZAP70) with the intracellular immunoreceptor-tyrosine-based activation motifs (ITAMs) of CD3. ITAMs are phosphorylated by the Src family PTK lymphocyte-specific protein tyrosine kinase (Lck). The Src kinase module is in charge of regulating the activity of the PTKs Lck and Fyn and ensures the TCR activation threshold. CD45 dephosphorylates inhibitory tyrosine of membrane-localized Src family kinases Fyn and Lck, previously recruited and activated by CD4 or CD8 co-receptors. Activated Fyn and Lck phosphorylates ITAMs on the CD3 *ζ* chains. This allows binding ZAP-70 to the ITAM, and activated ZAP-70 phosphorylates tyrosines on the adaptor protein LAT, which then attracts the phospholipase C (PLC-*γ*). Phosphatidylinositol-4,5-bisphosphate (PIP_2_) is the substrate for PLC-*γ*. PLC-*γ* hydrolyses the PIP_2_, generating two second messengers, inositol(1,4,5)trisphosphate (InsP_3_) and diacylglycerol (DAG). Whereas DAG activates the RAS/PKC pathway, binding of InsP_3_ to the InsP3R Ca^2+^-permeable channel, located on the endoplasmic reticulum (ER) membrane, mediates the release of Ca^2+^ from this store. Depletion of ER Ca^2+^ stores is sensed by the stromal interaction molecule 1 (STIM1) protein integrated in ER membrane, which in turn induces the opening of ORAI1 calcium release-operated calcium channels (CRAC) located in plasma membrane and Ca^2+^ influx [58, 59].

The process of conversion the Ca^2+^ signal into a biological response is assured by Ca^2+^-binding regulatory proteins, which together form an intricate network of feedback loops to control the location, amount, and effect of calcium influx. In T cells, CaM is considered as a major sensor and transducer of Ca^2+^ signals, and among CaM-binding signal traducers are Cn and Ca^2+^/CaM-stimulated protein kinases II and IV (CaMKII and CsaMKIV) [60].

Cn activation is foremost dependent on the intracellular Ca^2+^ concentration (reviewed in [61]). The Cn complex is composed of a catalytic subunit A (CnA) and a regulatory subunit B (CnB), tightly associated at resting conditions. CnB possesses four Ca^2+^-binding sites, two of which are of low and another two of high affinity. High-affinity sites are often referred to as structural ones and bind Ca^2+^ at nanomolar range, stabilizing the heterodimeric Cn structure. Little or no phosphatase activity is observed at [Ca^2+^]_i_ about 100 nM in resting cells. The low affinity sites with K_d_s in the micromolar range are considered as Ca^2+^ sensors. During signaling events causing a Ca^2+^ rise, binding of Ca^2+^ ions to these sites results in sequential conformational changes (partially active form), dissociation of the CaM-binding region, binding of CaM, and displacement of the autoinhibitory inhibitory peptide from the active site (fully active form). Additionally, Cn activity is regulated by Ca^2+^-independent endogenous inhibitor calcipressin [62].

In T cells, NFAT is considered as a major substrate for the Cn [63]. NFAT activity is regulated by its phosphorylation status. Under resting conditions, NFAT is highly phosphorylated. During activating events, dephosphorylation of multiple sites by Cn causes a conformational switch of NFAT protein that allows its translocation to the nucleus. Once inside the nucleolus, NFAT cooperates with multiple transcriptional partners and binds to specific DNA response elements to regulate the transcriptional program, which is specific for every cell type and for the stimulation pattern [64, 65]. Ca^2+^/Cn/NFAT signaling was initially described in mature T cells as a critical regulator of the TCR-induced IL2 gene transcription [66–68]. Later it was shown that this pathway regulates the expression of numerous genes, including cytokines, as well as genes, encoding proteins involved in the regulation of survival and proliferation, apoptosis, and cell cycle [64, 65].

It was estimated that 75% of all activation-regulated genes in T cells demonstrate dependence on the Ca^2+^ influx [53]. The changes in [Ca^2+^]_i_ have been detected as the cell grows and passes through G1, G1/S, and mitosis. Ca^2+^ not only operates upstream of the cell-cycle machinery by regulating the expression, activity, and/or location of the transcription factors that control expression of the G1 cyclins (FOS, JUN, MYC, CREB-ATF1 (activating transcription factor 1), and NFAT), but also acts more directly on the cyclins, CDKs, and/or their small protein inhibitors to regulate the assembly and activation of CDK complexes (reviewed in [56]).

Whereas initial T cell activation is related to short-term local [Ca^2+^]_i_ raise, subsequent events related to a new gene expression all require a sustained Ca^2+^ influx to maintain [Ca^2+^]_i_ at level higher than basal one in resting cells. In addition to CRAC channels, other Ca^2+^-permeable channels have been found in T lymphocytes and proposed as Ca^2+^ influx pathways during different physiological events (as discussed below).

### 4.2. Maturing Thymocytes

TCR signaling coordinates thymocytes maturation as well [6]. The survival of “correctly” developed thymocytes in the thymus depends crucially on the signaling trough pre-TCR [6]. The process of *β* selection is the first critical checkpoint related to the pre-TCR signaling [69]. Only DN3 cells that have productively rearranged a TCR*β*-chain, which can assemble with the invariant pre-T*α* and CD3 molecules to form the pre-TCR complex, are selected for further differentiation [70]. Expression of the functional pre-TCR in the DN3 promotes survival and proliferation (DN4) and following differentiation to the DP stage. Remarkably, transition of the DN to the DP demonstrates apparent independence of the pre-TCR on the ligand. The ligand-independent nature of pre-TCR signaling has been attributed to its localization closely to other signaling molecules in lipid rafts and to the relatively low signaling threshold of pre-T cells [71, 72]. Notch1 is considered as a probable candidate for this signaling molecule, because the interaction between Notch and its Delta-like ligand expressed at stromal cells indeed plays an essential role in enabling the autonomous signaling capacity of the pre-TCR complex [17, 73]. These findings provided a functional basis for the observed pattern of the Notch receptor expression and activation in developing thymocytes, since several reports have showed that levels of Notch1 and Notch3 expression and activity are significantly higher in the DN than in the DP thymocytes [10, 74–76].

T cell development from immature DP thymocytes to the mature CD4^+^ or CD8^+^ SP stage is also coordinated by the TCR signaling. The current working model suggests that the interaction strength between the TCR and the self-MHC complex determinates the destiny of maturated thymocyte. Little-or-none signal means that this thymocyte is unable to recognize self-MHC and will undergo death by neglect. A too strong signal will lead to a negative selection to avoid a generalized T cell aggression toward self-tissues. Only signals of intermediate range will culminate in survival (positive selection) [6].

TCR engagement activates the Cn/NFAT signaling not only during the activation of mature T lymphocytes, but also at the specific steps of thymocytes development (revised in [77]). By Cn inactivating during haematopoietic development, it was demonstrated that this signaling pathway plays an important, nonredundant role in the regulation of lymphocyte developmental checkpoints, in contrast to development of myeloid lineages [78]. More specifically, absolute requirement for Cn in positive but not in negative selection was demonstrated [79]. Early studies using a Cn inhibitor cyclosporine A (CsA) showed an impaired development of the DP thymocytes into SP [80, 81]. NFAT is upregulated during the T cells maturation [18, 19]. Disruption of NFAT in the DP thymocytes results in fewer SP cells and is associated with defects in the expression of the antiapoptosis protein Bcl-2 by DP cells [82]. Since NFAT activation is strongly dependent on the Ca^2+^ rise, involvement of transport systems in regulation of thymocyte maturation should be expected. Nevertheless, there are still few studies on this subject.

In experiments with mouse models, it was shown that the constitutive pre-TCR signaling induces the NFAT and NF-*κ*B activation, associated with an increased rate of the Ca^2+^ influx through the CRAC channels. Herewith, the biphasic nature of the cytosolic Ca^2+^ rise was observed, which differentially modulated the activities of the transcription factors NF-*κ*B and NFAT in developing T cells [28].

Recently, Lo and colleagues have investigated the Ca^2+^ signaling during positive and negative selection in the CD4^+^ MHCII-restricted T cells [83]. They demonstrated that negative selection induced a strong Ca^2+^ flux, and such a high Ca^2+^ peak might play a key role in inhibiting channel activity and decreasing transcript expression. On the other hand, a weaker yet more sustained Ca^2+^ flux was observed during positive selection. It was suggested that it may activate the Cn- and Erk-dependent pathways, leading to the survival and maturation. The authors consider that a sustained character rather than the magnitude of Ca^2+^ flux is the key function to support the positive selection. They identified a voltage-gated Na^+^ channel (VGSC), essential for a positive selection of CD4^+^ T cells. Pharmacological inhibition of the VGSC activity inhibited sustained Ca^2+^ influx induced by positive-selecting ligands and* in vitro* positive selection of CD4^+^ but not CD8^+^ T cells.

Interestingly, there are indications that the positive selection of CD4^+^ T cells may involve somewhat more intense and long-lasting signals that are required for the positive selection of CD8^+^ T cells [84]. Melichar and colleagues reported a distinct temporal pattern of the T cell receptor signals during positive versus negative selection in CD8^+^ cells* in situ* [85]. However, in contrast with studies carried out on CD4^+^ cells [84], they found that brief serial signaling events, which were separated by migratory periods and low cytosolic Ca^2+^, correlated with the positive selection of MHCI-restricted thymocytes, whereas sustained signaling and arrest of thymocytes were associated with negative selection [85].

It is widely accepted that Ca^2+^ entry through CRAC channels is the main pathway to increase intracellular Ca^2+^ concentration in the peripheral blood T cells [58]. But CRAC seems not to play a central role in Ca^2+^ signaling during T cell maturation because T cell positive selection is normal in multiple separate knockouts of STIM and ORAI [86]. Loss-of-function STIM1 mutations were also reported in human patients [87]. Clinically, they demonstrated severe immunodeficiency with susceptibility to viral and bacterial infections, but practically normal T cell repertoire. The latter clearly indicates that the T cell maturation was not greatly affected. But, as expected, their T cells were unable to generate Ca^2+^ rise in response to antigenic stimuli.

Thus, regulation of multiple decision steps in thymocyte development is coupled to a complex modulation of Ca^2+^ fluxes, but CRAC channels do not seem to necessarily play the central role. It was proposed that nonstore operated Ca^2+^ channel(s) which might operate independently of STIM and ORAI may be involved, or alternatively, CRAC channels play an important but redundant role [83, 88]. There are some evidences in favor of this hypothesis. For example, deletion of TRPM7 Ca^2+^/Mg^2+^-permeable channel with an intrinsic kinase activity results in a block in thymocyte development at the DN stage [89]. Thereby the presence of “unusual” ion channels reported in leukemic cell lines may reflect both normal developmental thymic events and leukemogenesis.

### 4.3. T-ALL

It was the subject of long time debate, whether the contribution of Ca^2+^ signaling is imperative for the tumor growth progression. Conventional view supported the idea that malignant cells are much less dependent on Ca^2+^ during the proliferation than healthy cells, and even loss of proliferative dependency on Ca^2+^ was considered as a hallmark of malignant transformation (reviewed in [56]). Although relative independence on Ca^2+^ may occur in some type of cancers, the situation in general is much more complex. The question rather should be discussed in terms of the Ca^2+^ signaling deregulation, where some elements of the Ca^2+^ toolkit in transformed cells are downregulated whereas others are upregulated. Monteith and colleagues have undertaken a thorough analysis of available data, concerning variations in the expression and activity of some Ca^2+^ channels and pumps in tumors and cancer cell lines. They did not reveal any uniform profile characteristic for cancerous cells [90]. Rather, they point out to some potentially predictable consequences of the trends. They also provided available data on the aberrant location of Ca^2+^ channels in many types of tumors, which could change the nature of the Ca^2+^ signal and a subsequent biological response.

Ca^2+^ homeostasis controls various cellular processes, which are relevant to the tumorigenesis, such as proliferation, apoptosis, gene transcription, and angiogenesis (see for review [90]). Ca^2+^ is a key regulator of proteins, implicated in the cell cycle regulation: Ras, immediate early genes in G0/G1 transition, retinoblastoma (Rb) protein in G2. In addition to cell cycle regulation, Ca^2+^ is implicated in cellular motility, which in turn contributes to the tumor invasion and metastasis. Ca^2+^ was shown to be an important regulator of genomic stability and transcription, critic events in leukemogenesis. There are limited studies that specifically address possible alterations in different elements of Ca^2+^ toolkit and Ca^2+^ signaling in tumorigenesis. Therefore it looks that question about these kinds of alterations should be addressed specifically for every type of tumors.

Taking into account that the TCR signaling related to the Ca^2+^ rise and Cn activation is a central coordinator of T cell physiology, we will analyze the possibility of contribution of this pathway to T cell malignancy.

Proliferation of leukemic cells is obviously antigen-independent. But being derived from the T-lymphoid precursors, arrested at different early stages of the development, T-ALLs demonstrate an astonishing heterogeneity in their TCR/CD3 phenotypes [91, 92]. Apropos, T-ALL classification by EGIL (European Group for the Immunological Classification of Leukemias), is based on the presence of CD3 and TCR chains [93]. The cytoplasmic and then membrane expression of CD3 is an early event in the T cell ontogeny [94]. Then the presence of CD3, either at the surface (sCD3) or in the cytoplasm (cCD3), is a determinative feature of these malignances [94]. TCR genes rearrangements are found in a majority of the T-ALL; the presence of TCR chains at the surface or in cytoplasm is mostly characteristic for mature stages [95]. Accordantly, T-ALLs are distributed in five immunophenotypic subtypes: pro-T-ALL (TI), pre-T-ALL (TII), cortical-T-ALL (TIII), and mature *αβ* or mature *γδ* (TIV) [93, Figure 2].

TI leukemias do not possess the TCR chains [2]. In some animal models like E2A-deficient mice, defects that prevent the pre-T cell antigen receptor expression even tend to accelerate the Notch-dependent lymphomagenesis [96]. Then the question arises, whether the pre-TCR or TCR is involved in the neoplastic transformation in other more mature T progenitors. It seems logical that the pre-TCR/TCR requirement for leukemogenesis greatly depends on the context and other signaling pathways involved in process. Accordingly, the role for the pre-TCR/TCR was studied in diverse mouse models, where leukemogenesis was provoked by abnormalities in different signaling pathways. It has been observed that the pre-TCR assists leukemogenesis, driven by the Notch activation [97–99], c-Myc overexpression [100], or Ikaros deficiency [101]. In contrast, other T cell leukemia mouse models, such as eg* Trp53-* or* ATM-*deficient mice, do not show such pre-TCR dependency [102, 103]. Some of these studies are described in more detail below.

Bellavia and colleagues used transgenic mice with upregulated constitutively active intracellular domain of the Notch3, which is ordinarily downregulated as thymocytes maturate [97]. The mice developed early and aggressive T cell neoplasias with features of immature thymocytes, including expression of the pT*α*, a defining component of the pre-T cell receptor, known to be a potent signaling complex provoking thymocyte survival, proliferation, and activation. Deletion of the pT*α* in Notch3 transgenic mice abrogates tumor development, indicating a crucial role for the pT*α* in the T cell leukemogenesis. In addition, the analysis of 30 samples, derived from children with T-ALL, demonstrated expressions of Notch3 and its target gene HES-1, as well as of pT*α* transcripts. Remarkably, the expression of all these genes was dramatically reduced or absent in the remission. In another clinical report, SCL overexpression was invariably associated with a high TCR expression in childhood T-ALL [1].

Similarly, pre-TCR expression was demonstrated to cooperate with TEL-Jak2 to transform thymocytes and induce rapid T-ALL [99]. However, in the pre-TCR-deficient TEL-JAK2 mice, the T cell leukemogenesis was only delayed but not canceled [104]. In Notch-dependent T-ALL, pre-TCR signaling was required to condition mice for the Notch-dependent transformation but it was not required to sustain the malignant growth of the T-ALL [98]. Since the pre-TCR signaling is associated with a proliferative burst of thymocytes, accompanying differentiation stages [28], it was suggested that the pre-TCR-assisted proliferation in preleukemic cells increases the probability to acquire secondary oncogenic events, ultimately leading to a clonal disease [99]. Importantly, TCR expression induces the leukemic cell expansion in secondary lymphoid organs indicating the importance of the TCR-related signaling for the motility of leukemic cells [99].

Further studies of the pre-TCR signaling in leukemogenesis were designed to reveal the importance of the associated CD3 molecules for the leukemogenesis. The experiments were based on the fact that the pre-TCR and TCR require association with the Cd3*ε* for signaling [105]. Therefore, the Cd3*ε*-deficient cells have nonfunctional pre-TCR/TCRs. As it was shown, the absence of the pT*α* chain only slightly delays the appearance of the TAL1/LMO1-induced T-ALL in mice, while* CD3ε*-deficient mice do not develop the TAL1/LMO1-induced T-ALL [106]. Then it was concluded that pT*α* chain seems to play a minor role, but the* CD3ε*-mediated signal transduction pathway is essential for the transformation process. Similar results were obtained with another, namely, SCL/LMO1-induced T-ALL, using transgenic mice as a model [33]. They show that mice with SCL/LMO1 upregulation developed the Notch1 activation and T-ALL with a 100% penetrance, whereas, in strain with an additional Cd3*ε* deficiency, the penetrance of the disease was decreased by 48% and the median survival significantly increased. It was suggested that SCL, LMO1, and Notch1 together with an active pre-TCR/CD3 might represent the minimum set of complementing events for the transformation of susceptible thymocytes [33].

Immunophenotyping of frequently used T cell leukemia cell lines revealed high heterogeneity in the TCR expression [91]. It was suggested to consider the particular differentiation stage of each individual cell line, while using the T-cell leukemia lines as models for malignant or normal T cells.

Altogether the data available to the moment indicate that the pre-TCR/CD3 signaling accelerates the T cell leukemogenesis, being involved in the proliferation and in the expansion of leukemic cells to secondary lymphoid organs.

Cn is sustainably activated in T lymphoid malignances, both in animal models and biopsies from human lymphomas [43, 107–109]. The role of Cn activity in the pathogenesis of T-ALL was demonstrated in well-designed experiments with the usage of two different mouse models, related to human T leukemias [43]. In one model, bone marrow cells were retrovirally transduced with a construct, encoding the activating intracellular Notch1 domain. In a second model, transgenic mice expressed the TEJ/JAK2 fusion protein. In both models, mice developed the T-ALL, with a constitutive dephosphorylation of the NFAT in leukemic cells, indicating an aberrant Cn activation. Mice treatment with the Cn inhibitors CsA and FK506 resulted in disease remission with the hematopoiesis restoration [43].

Increased Cn/NFAT activity by the Notch signaling was shown to involve a downregulation of calcipressin through the Hes1-dependent mechanism [110]. As far as the Hes-1 is upregulated in T leukemias through the Notch1, this mechanism was proposed to be involved in the leukemogenesis [62, Figure 2]. Few reports have described different mechanisms of a sustained Cn activation due to the gain-of-function mutation in the* CnA* in T or B lymphoma-derived cell lines [111, 112]. In the EL4 murine T lymphoma cells, a missense mutation changed an evolutionary conserved aspartic acid to the asparagine within the autoinhibitory domain of the* CnA*α* gene [111]. This substitution leads to the generation of a mutant CnA*α*,* hypersensitive to Ca^2+^ [111]. But still, the elevated [Ca^2+^]_i_ is an indispensable factor to maintain the Cn activity, since mutations resulting in a persistent Cn activation, independent of Ca^2+^, were never reported.

As was already mentioned earlier, the NFAT activation is a hallmark of the T-ALL. While activated mutations of NFAT genes have been never observed in human cancers, the aberrant NFAT signaling in tumors was suggested to involve either its overexpression and/or hyperactivity (reviewed in [113]). NFAT hyperactivity in the T-ALL is likely to be related to the Notch-dependent Cn upregulation (Figure 2). NFATc1 nuclear localization or dephosphorylation of both NFATc1 and NFATc2 were found in primary tumor samples and cell lines, derived from a patient with an aggressive T-cell lymphoma. Moreover treatment of these cell lines with CsA triggered the cell cycle inhibition and induced apoptosis [43].

## 5. Neglected Pathway in the T Cell Physiology: Lymphoid Cholinergic System Is Upregulated in Leukemias

Calcium mobilization, following the TCR ligation during the T cell activation is essential but is not the only way of the Ca^2+^ rise generation in T lymphocyte. Less considered pathway is related to a nonneuronal lymphoid cholinergic system. Lymphocytes possess all components of independent cholinergic system that include the acetylcholine (ACh), choline acetyltransferase (ChAT), its synthesizing enzyme, and both muscarinic (mAChR, M1–M5) and nicotinic (nAChR) ACh receptors [114–116]. Human T lymphocytes produce a small quantity of Ach and up-regulate the ChAT and mAChR mRNA expression in a response to the TCR activation. Ligand binding to the PLC-coupled M1, M3, and M5 mAChRs induces rapid increases in [Ca^2+^]_i_ with Ca^2+^ oscillation via the IP_3_-evoked Ca^2+^ rise from the intracellular store. This pathway was suggested as an amplification mechanism to increase the IL2 and IL2R production [117] and the c-fos expression [116] during the TCR stimulation. In addition, M1 is known to play a critical role in the differentiation of CD8^+^ cells into the CTL [118]. Emerging evidence indicates that mAChRs may be implicated in the regulation of the cell proliferation and cancer progression in leukemogenesis [119]. As for the T cell leukemias, the Ach production was shown to be drastically increased in different T-ALL-derived cell lines, when compared to the resting mononuclear cells [120]. It could be suggested that a sustained Ca^2+^ influx in leukemic cells may be maintained by elevated Ach production and autocrine stimulation through the mAChRs ligation.

## 6. What Do We Know about the Relationship between T Leukemia-Related Mutations/Pathways and Ion Channel Expression and Activity?

Taking into account the purpose of the present review, we have undertaken the bibliographic search of the data on possible involvement of transcriptional elements, misregulated in the T-ALL, in the ion channel expression and activity.

To identify TAL1 direct target genes, Palomero and colleagues have undertaken the chromatin immunoprecipitation experiments with antibodies raised against the TAL1 in Jurkat CD4^+^ cell line as a model [121]. It was shown that the TAL1 binds to promoters of 71 target genes, encoding proteins important for many vital cellular processes. The list of target genes includes receptor and surface molecules, intracellular signal transduction elements, transcription factors, and DNA-associated proteins, proteins that participate in the DNA reparation, vesicular trafficking, drug resistance, secreted molecules and growth factors, ion channels, and transporters. Among ion channels and transporters which represent a significant portion of direct targets for the TAL1 the authors specifically indicated the CHRNA5 (subunit alpha-5 of the nicotinic acetylcholine receptor), ACCN2 (Amiloride-sensitive cation channel 2), CACNG4 (gamma-4 subunit of the L-type voltage-dependent calcium channel), KCNJ9 (G protein-activated inward rectifier potassium channel 3), SLC4A11 (sodium bicarbonate transporter-like protein 11), and OKB1 (organic cation transporter). The presence of other transcriptional factors as TAL1 target proteins suggests the existence of a very complex TAL1-dependent transcriptional network in the T-ALL with aberrant expression of a number of proteins implicated in different cell processes. Importantly, it was shown that TAL may act as an activator or as a repressor for target genes. Further experiments with other T leukemic lines (MOLT) and primary T-ALL samples demonstrated high levels of variation in the expression profiles of TAL1 target genes.

Notch 1 activation, being a hallmark of many types of the T-ALL, is also involved in the pathophysiology of other cancers. Then we undertook a search for data on the possible relationship between the Notch1 activation and ion channel expression. For example, aggressive and malignant state of glioblastoma multiforme (GBM), the most frequent and incurable type of the brain tumor of adults, was shown to be related to an increased activation of the Notch1 provoked by hypoxia [122]. Notch1 activation in turn induced the expression of transient receptor potential 6 (TRPC6) Ca^2+^-permeable channels in primary samples and cell lines derived from GBM. Functionally, TRPC6 caused a sustained elevation of the intracellular Ca^2+^ coupled to the activation of the calcineurin-related NFAT pathway. TRPC6 was shown to be required for the development of the aggressive tumor phenotype, because a knockdown of the TRPC6 inhibited the glioma growth, invasion, and angiogenesis. Notch-dependent transcription of the TRPC6 was reported in pheochromocytoma PC12 cells [123]. Interestingly, TRPC6 mRNA was also found in the T-ALL cell line Jurkat, in contrast to T cells obtained from healthy donors, but the question about its relation to aberrant activation of Notch1 and NFAT was not addressed yet [124].

## 7. Nondifferentially Expressed Genes in T-ALL Signaling: Ion Channels May Be Involved

Traditionally, the T-ALL diagnostics and corresponding therapeutic strategies are based on the differential expression (DE) of genes, that occurred in the T-ALL patients as compared to healthy persons. However, as it was recently pointed out by Maiorov and colleagues (2013), the expression of some genes might not vary in the T-ALL, but, instead, these genes may be interconnected with highly differentially expressed ones [125]. They have proposed a network-based approach instead of the expression-based one for better understanding and management of the T-ALL and identified 19 significant subnetworks that represent clusters of functionally related, both DE and non-DE, genes. So non-DE genes code the proteins that in pathologic conditions may be involved in signaling networks different from those they normally belong to. It was proposed that non-DE genes could be essential in the interconnection of numerous DE genes and play important roles in malignant transformation of the precursor T cells. Purinergic receptor P2RX7, complement component C9, plasminogen, Ca^2+^-binding protein CHGA, and peptide hydrolase MEPIA were pointed out among the non-DE genes in T-ALL.

## 8. Ion Channels in T Cell Physiology

Tumor cells survival and proliferation, activation, differentiation and malignant progression, invasiveness/migration (via volume regulation, polarization, cytoskeleton, and extracellular matrix reorganization), and, at last but not the least, the resistance to anticancer therapies, all these critically depend on the function of ion channels. T lymphocytes and leukemic T cells bear authentic orchestras of K^+^-selective, Ca^2+^-selective, and nonselective cation and anion channels. Wherein, their local combinations one with another and with other signaling components form a highly specific microenvironment, essential for cellular performance. The first purpose of this chapter is to give an overview of ion channels, expressed in T-cells, along with their known functions and giving emphasis to those, which are differentially expressed in healthy and leukemic cells. The second purpose is to revise the channel-to-channel functional communications, especially those related to a formation of specific Ca^2+^ signal. The third is to present, whenever it is possible, the structural and functional view on membrane signaling complexes, involving an ion channel as a core element.

Potassium- (K^+^-) selective channels reported up to the date in healthy or neoplastic lymphoid cells belong to several families: voltage-gated (K_v_), Ca^2+^-activated (K_Ca_), and tandem-pore domain (K2P) channels. K^+^ channels may act via their ionotropic function or via noncanonical nonconducting mechanisms, that is, via direct interaction with other membrane or cytosolic proteins. Channels' mediated K^+^ transport causes a change of membrane voltage, affects a driving force for Ca^2+^ influx, and, via changes of intracellular K^+^, regulates the cell volume (in parallel with the activity of anion channels, see below) and, more specifically, affects the activity of the intracellular machinery, for example, activation of caspases in the course of apoptosis [126–128]. Membrane potential affects the cell cycle progression. Whereas G1/S transition is associated with a hyperpolarization (high K^+^ conduction), the G2/M one occurs with a depolarization and low K^+^ conductance [129]. Blockers of K^+^ channels cause G1 arrest [130]. Also volume changes during cell cycle, for example, volume decrease during the M-phase prior to cytokinesis, requires a K^+^ efflux via voltage-gated (e.g., K_V_10.2) K^+^ channels [131]. G1/S progression and G2/M transition also require Ca^2+^ bursts. The former includes activation of CDKs and culminates through phosphorylation of Rb1 in activation of E2F transcription factors. During progression of the G1 phase several processes like expression of AP1 (JUN and FOS) and CREB transcription factors, as well as regulation of cyclins, are calmodulin dependent, whereas transport of the transcription factor NFAT to nucleus requires the calcineurin-mediated dephosphorylation [56]. In T cells the Ca^2+^ signaling is critically dependent on the activity of partner K^+^ channels as will be discussed below.

### 8.1. K_v_1.3

**Figure 3:**
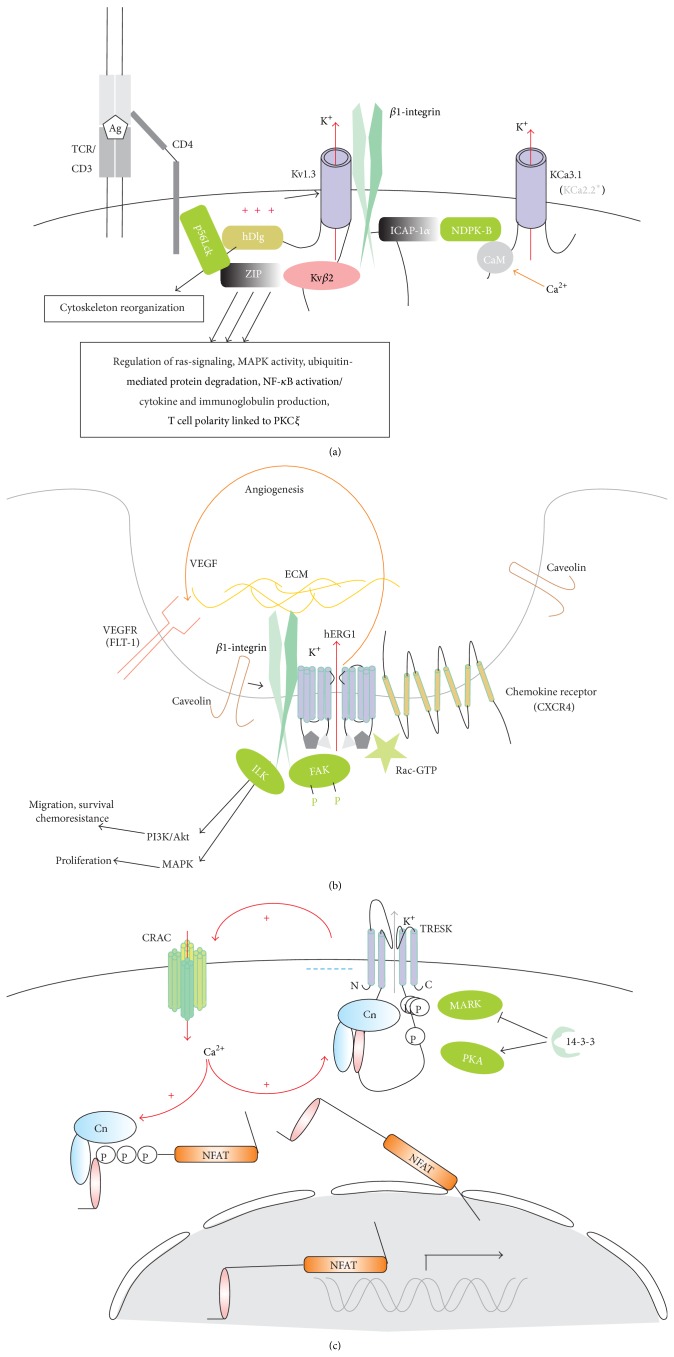
K^+^ channels and their partners in the plasma membrane of T-cells. (a) Kv1.3 channels are activated and inactivated by the membrane depolarization. Its cytosolic N terminus binds to auxiliary, NADP binding *β*-subunit Kv*β*2, possessing oxidoreductase activity, and sensing cell metabolism and redox state. It also mediates trafficking of members of Kv1 subfamily to plasma membrane. Beta-subunit via multifunctional adapter protein ZIP is linked to p56Lck, phosphotyrosine kinase in a phosphotyrosine-independent way. ZIP regulates a variety of intracellular processes as indicated. In turn, p56Lck is connected via PDZ-domain (hDlg) to the last three residues at the C-terminus; binding of hDlg to a kinesin motor protein (GAKIN) and provoking cortical cytoskeleton reorganization. Open state Kv1.3 conformation favors its binding to *β*1-integrin and the latter via integrin cytoplasmic domain associated protein (ICAP1-1a) and nucleoside diphosphate kinase NDPK-B (activating KCa3.1 via phosphorylation) communicates to intermediate conductance Ca^2+^-activated K^+^ channel KCa3.1 (substituted for small conductance KCa2.2 in Jurkat cells). Kv1.3 can promote clustering of all aforementioned interacting proteins plus CD4 in the immunological synapse. A colocalization of Kv1.3, KCa3.1, and CRAC channels is essential for synapse stability, via local K^+^ (K^+^ accumulation in the cleft, membrane depolarization) and Ca^2+^ signaling (for a further discussion, see [127]). Linking of Kv1.3 to integrin allows a bidirectional signaling, when K^+^ channel gating transmits to cell adhesion (inside-out signaling) and, vice versa, integrin-mediated external signal (outside-in) may be traduced to intracellular events via Kv1.3 and its interacting proteins. (b) hERG1 channels physically interact with *β*1-integrin receptors and are concentrated in caveolas, a type of lipid rafts, enriched of caveolins. Binary complexes of hERG1 with *β*1-integrin are typical for cancer cells. “Outside-in” signaling: hERG1 activation links *β*1-integrin adhesion to fibronectin (in the extracellular matrix, ECM) to the tyrosine phosphorylation of FAK and activation of small GTPase (Rac); both are coprecipitated with the channel protein. Caveolin association with *β*1-integrin promotes the channel activation [132]. FAK and ILK are primary targets for integrin-mediated cell adhesion, which transmits to the activation of MAPK, PI3K, and small GTPases. In leukemias trimolecular complexes can be also formed. In AML the third partner may be VEGF receptor, with an autocrine (hERG1-dependent) mechanism via VEGF secretion [133]. In ALL cells, hERG1/*β*1-integrin complex interacts with 7-TM domain (CXCR4, chemokine) receptors, stimulating signaling via ILK to Akt. Activation of this complex can be achieved via integrin engagement or CXCR4 chemical activation; ILK activity is suppressed by hERG1 specific block [134]. FAK (focal adhesion kinase), PI3K (phosphoinositide-3 kinase), VEGF (vascular endothelial growth factor), ILK (integrin linked kinase), and Akt (protein kinase B). (c) Tandem-pore domain K^+^ channel TRESK is activated by dephosphorylation by calcineurin (Cn) and suppressed by a phosphorylation of certain serine residues within the cytosolic loop between transmembrane domains II and III by protein kinase A (PKA) and a second kinase, likewise MARK, or microtubule affinity-regulating kinase [135]. Cn is activated either directly by Ca^2+^, Ca^2+^-CaM, or Ca^2+^-CaM-dependent kinase. Ca^2+^ influx in T-cells is mainly mediated by CRAC. Thus, TRESK and CRAC potentiate the activity of each other: TRESK generates driving force for Ca^2+^ influx by CRAC via membrane hyperpolarization, and CRAC shifts TRESK phosphorylation status to the dephosphorylated active form via Ca^2+^-Cn. Sustained increase of Ca^2+^ via Cn dephosphorylates NFAT and allows its entrance through a nuclear pore, hence activating genes transcription. Both TRESK and NFAT contain a characteristic Cn-docking site (PQIVID for TRESK and PxIxIT for NFAT), thus coupling the Ca^2+^ signal positive feedback loop to genes expression via Cn activity. 14-3-3 protein docking to the phosphorylated S264 protects it from the dephosphorylation; additionally, 14-3-3 protein inhibits the second kinase.

K_v_1.3 is the only one of 40 of the K_v_ family members known in mammals [136], which is expressed in healthy human T cells; in murine T cells, K_v_3.1, K_v_1.1, K_v_1.2, and K_v_1.6 were found in addition [126]. K_v_1.3 is steeply activated by a depolarization with a half-activation at about −30 mV and inactivates up to by 95% in even more steeply voltage-dependent process, with a midpoint around −50 mV. At resting membrane potentials (−40–−50 mV) only a tiny fraction of K_v_1.3 channels remains open at a steady state [137–139]. Thus, lower membrane potentials down to K^+^ equilibrium, which are observed at the G1/S transition in the cell cycle, require the activity of some voltage-independent K^+^ (e.g., K_Ca_ or K2P) channel(s), open at this voltage range. K_v_1.3 channel at its C-terminus forms a functional complex with the *β*1-integrin, the PDZ-domain protein SAP97, linked to the p56Lck kinase and an adapter ZIP protein; the N-terminus in turn could bind a K_v_ *β*2 subunit (redox sensing), which may eventually interact with ZIP protein [140, Figure 3(a)]. Channel opening, provoked by membrane depolarization, stimulates functional and physical interactions between K_v_1.3 and *β*1-integrin moieties and activates the integrin function, adhesion, and migration, whereas specific K_v_1.3 channel blockage prevents the integrin signaling [141]. Functions of the K_v_1.3 in T cells include but not restricted to (1) control of membrane potential against a depolarization challenge, depending on the T cells subset [142, 143]; (2) regulatory volume decrease (RVD), together with VSOR [144, 145], and apoptotic volume decrease (AVD) [146, 147]; (3) support of a sustained Ca^2+^ influx by CRAC, generation of Ca^2+^ oscillations (together with K_Ca_3.1 and TRPM4). A sustained, lasting over hours, Ca^2+^ increase, which is primordial for new gene expression, is triggered in T cells via the Ca^2+^/calcineurin/NFAT pathway [55]. A block of both K_v_1.3 and K_Ca_3.1 tends to abolish Ca^2+^ oscillations, with an impact on T cells proliferation [148]. In Jurkat leukemic T cells, selective inhibition of K_v_1.3 abolished oscillations of the CRAC-mediated Ca^2+^ entry but not the average Ca^2+^ entry [149], which likely depends more on the K_Ca_ channels activity (see below). Obviously, CRAC and K_Ca_3.1 activities are linked in a feedforward manner, so that the activation of K_Ca_3.1 by Ca^2+^ influx via CRAC will hyperpolarize the membrane, increasing CRAC-mediated Ca^2+^ influx, and so forth. To reverse this Ca^2+^ increase, additional mechanisms are required which may be CRAC inactivation by Ca^2+^ [150] and/or membrane depolarization, caused by unique Ca^2+^-impermeable member of the TRP family, the TRPM4 (see below). Kv1.3 is a predominant (several hundred copies per cell) K^+^ channel in naïve human T cells, which functionally express just few K_Ca_3.1. K_v_1.3 plays a very essential role in the lymphocyte activation and associated Ca^2+^ signaling and IL-2 production. Upon the activation, differential behavior is observed in diverse T cell subsets. In activated T_EM_ (effector memory) cells K_v_1.3 is selectively upregulated (highK_v_1.3 : lowK_Ca_3.1 phenotype); on the contrary, in T_CM_ (central memory) cells, Kv1.3 only modestly upregulated, whereas K_Ca_3.1 dramatically (by 1.5 order of magnitude increase), producing lowK_v_1.3 : highK_Ca_3.1 phenotype [151–154]. Accordingly, specific inhibition or silencing of the K_v_1.3 decreases the proliferation of T_EM_ without significant effect on T_CM_ and naïve cell population [155]. Immunosuppression can be achieved via the Kv1.3 specific inhibition and resulting depolarization, which attenuates the Ca^2+^ influx via CRAC. Selective K_v_1.3 suppression was only efficient upon Ca^2+^-dependent lymphocyte activation and not in the cases of CD28 or IL-2-induced activation, which is independent on the intracellular Ca^2+^ rise [156]. Curiously, when expressed in heterologous system, K_v_1.3-mediated cells proliferation was unaffected in a poreless mutant (albeit sensitive to supposedly sole open-pore blockers, MgTx and PAP-1) but abolished in a mutant with altered voltage gating [157]. It appears then that the conformational change of K_v_1.3 protein upon the channel opening may be sufficient for an efficient signaling, without involving a K^+^ flux or membrane polarization. Such conformational coupling may be mediated by a close association of Kv1.3 channels with *β*1-integrin [141, 158].

Comparison of the K_v_1.3 channels density in human peripheral blood resting T cells [137, 138, 141, 153, 159–163] and Jurkat lymphoblasts [139, 147, 149, 164–167] gives mean values of 380 and 215 active K_v_1.3 copies per cell, respectively. In activated T cells, depending on subpopulation, the number of K_v_1.3 channels could increase, modestly or very significantly, up to 1800 copies per cell [153]. Taking an approximately 3-fold larger membrane surface in Jurkat lymphoblasts into account, the K_v_1.3 density, expressed as number of channels per unit area, is substantially lower in Jurkat cells. Yet, bearing in mind a complex Kv1.3 regulation within a signalosome, with all or some of interacting proteins involved [140] one may wonder, should such regulation be equally or more important than just a variation of the K_v_1.3 copies numbers. Signaling complex, presented in Figure 3(a), exists within an immunological synapse. Thus, in case of leukemic cells, another question is whether there are alternative regulatory proteins, which interact with K_v_1.3 in these cells and what is the outcome of such interaction. Regulation of K_v_1.3 by p56Lck kinase [164, 165] as well as a colocalization with TCR/CD3 [168] is preserved in Jurkat cells. We are not aware, however, of any evidence pro or contra the functional interaction of K_v_1.3 with integrins in leukemic T cells. It should be noted that p56Lck kinase mediates oxygen sensing by K_v_1.3; hypoxia is a common condition within a tumor tissue and tends to suppress the K_v_1.3 channel activity, thus, T-cell proliferation under conditions of O_2_ deprivation [169]. Healthy human leucocytes and Jurkat cells express a second type of *β*-subunit, KCNE (1 to 5). KCNE 4 but not 2 physically interacts and substantially modifies the Kv1.3 gating, decreases the channel surface expression, and impairs channel's targeting to lipid rafts [170]. Upon Jurkat activation with fetal bovine serum (FBS), KCNEs 2 and 4 were upregulated, but the same KCNEs were downregulated upon Jurkat activation by PHA [171]. Voltage gating of Kv1.3 was differentially modulated by the lipid rafts disintegration in healthy T cells [138] and Jurkat cells [139], suggesting a different microenvironment.

Here we will mainly discuss the roles, played by plasma membrane ion channels. However, several intracellular channels and transporters, expressed in nuclear, ER, and mitochondrial membranes, especially the latter, play important roles in cancer cells proliferation and survival [172]. In particular, some K^+^ channels have dual expression, in the plasma and intracellular membranes. K_v_1.3 channel, discussed above, is expressed both in plasma and inner mitochondrial membranes. Plasma membrane K_v_1.3 may be selectively blocked by membrane-impermeable toxins, whereas mitochondrial K_v_1.3 is only blocked by membrane-permeable drugs; blockage in the latter case induced apoptosis in cancer cells, including leukemic ones [172]. In addition, mitoK_v_1.3 interacts directly with Bax in Jurkat cells. This gives rise to a sequence of events, including the hyperpolarization of the inner mitochondrial membrane, ROS generation, and cytochrome c release from mitochondria, thus, promoting the intrinsic apoptotic pathway [173].

### 8.2. hERG1

hERG1 (human ether-a-go-go-related gene, Kv11.1) channels are normally expressed in excitable cells, primarily, in neurons and heart [174]. In heart it encodes a rapid delayed rectifier (I_Kr_) K^+^ current, activating at membrane depolarization and mediating action potential repolarization. It has very peculiar biophysical properties, possessing a rapid inactivation at positive potentials and a rapid relief of inactivation at negative ones. Therefore, the steady-state I/V relationship for this channel is bell-shaped, with a peak around −10 mV, where also the channel's open probability reached its maximum. Yet, channel's deactivation (a process of the reversal of activation) at negative potentials is slow (hundreds of milliseconds). Thus, at the peak and plateau of the action potential hERG1 conductance is greatly reduced due to the inactivation; it started to increase and reached its maximum at phase 3 repolarization [175]. In conclusion, hERG1 channels need to open first upon the action potential firing (depolarization), but they act (contribute) mainly on the way back, when a rapid relief from the inactivation followed by a slow deactivation allows a large K^+^ efflux, promoting further repolarization. However, the midpoint of the hERG1 activation is around −30 mV, so that it possesses a significant activity down to −40 mV, which is close to resting potential in some nonexcitable cells, including lymphocytes. hERG1 is undetectable or expressed at very low levels in healthy immune cells. But it is frequently abnormally overexpressed in many cancer types, in particular, in acute lymphoblastic as well as in chronic lymphocytic (CLL) and both in acute and chronic myeloid (AML and CML) leukemias [134, 176–178]. hERG1 was found in self-renewing population of leukemic stem cells [179] and also in healthy precursor CD34^+^ cells from peripheral blood, but only after cytokine stimulation [176]. Importantly, unlike in healthy cells, in tumors hERG1 is often found in functionally binary complexes with *β*1-integrin, or even in triple protein complexes (Figure 3(b)), thus linking external to intracellular signaling and vice versa [127, 180]. hERG1 can increase oncogenic potential in leukemias by affecting one of several ways facilitating leukemogenesis: (1) balance between proliferation and cell death; (2) invasiveness, depending on the fine balance between adhesion and motility, the latter being in turn dependent on polarized volume changes; (3) resistance to chemotherapy [127, 180]; (4) angiogenesis, via secretion of vascular endothelial growth factor (VEGF) and positively feedbacked microvesicles release [181, 182]. hERG1 surface expression depends on the interaction of different isoforms; presence of hERG_uso_, which form heterotetramers with other hERG1 isoforms that resulted in an arrest of hERG1 in endoplasmic reticulum, with a further degradation, thus, substantial decreasing hERG1-mediated currents across the plasma membrane [183]. Cancer cells, including leukemias, often express a truncated hERG1b isoform, lacking the oxygen-sensing domain. It forms heterotetramers with full length isoform hERG1 and the higher is hERG1/hERG1b ratio, the lower is voltage threshold for the channel activation, and more hyperpolarized is the membrane potential. Upregulation of hERG1b during the S-phase consequently resulted in more depolarized membrane potential thus, assisting the cell cycle progression. On the other hand, at hypoxic conditions, which are typical for tumor progression, increase of hERG1/hERG1b ratio could hyperpolarize membrane potential, reducing K^+^ loss, thus handicapping AVD and the apoptosis [184]. Although there is specific pharmacology against hERG1, its usage to treat hERG1 in leukemias is handicapped by the fact that cardiac hERG1 may be also affected, producing long QT-syndrome, which can eventually cause a fatal fibrillation. Yet, drugs only affecting the open channel state may be given a preference compared to blockers, acting also in the inactivated state, in case of hERG1 R-roscovitine and E4031, respectively [174]. In heart, hERG1 opens only shortly, at phase 3, whereas most of time hERG1 is either deactivated (at resting potential) or inactivated (at depolarized potentials). However, to properly address anti-hERG1 therapy to T-ALL treatment more studies on this type of leukemia are required. Except a single report on CEM cell line [177], hERG1 function and signaling were investigated on all types of leukemias but not on T-ALL. In particular, we and others were unable to detect any measurable hERG1-mediated currents in Jurkat cells.

### 8.3. K_Ca_3.1

K_Ca_3.1 (IKCa1), intermediate conductance K_Ca_ channel, is voltage independent and is activated by Ca^2+^-CaM (CaM bound to C-terminus) with an apparent K_d_ = 300 nM [126]. K_Ca_3.1 also requires the phosphatidylinositol-3 (PI(3)P) for its activity, albeit it is not clear how this activation achieved; the effect on the channel is indirect [185]. Downstream to the PI(3)P is a reversible phosphorylation of channel's tyrosine 358; silencing of respective kinase (nucleoside diphosphate kinase B or NDPK-B) or phosphatase (histidine phosphatase PHPT-1) caused a suppression or activation of the K_Ca_3.1 current and efficiently modulates, in opposite way by NDPK-B or PHPT, Ca^2+^ influx and CD4^+^ T cells proliferation after stimulation [186, 187]. The activation of K_Ca_ provokes a stable hyperpolarization down to equilibrium potential for K^+^, E_K_ [188]. This hyperpolarization is indispensable for T cells, apparently lacking depolarization Ca^2+^ channels (see below), where CRAC (SOCE) is believed to be a central Ca^2+^ influx channel. Although resting T cells express only few copies of K_Ca_3.1, it is rapidly upregulated upon the activation and its expression level rises about 25-fold [189]. K_Ca_3.1 channel is involved in various cellular processes, namely, activation via TCR-engagement and stabilization of the immunological synapse; control of membrane potential, RVD, cell migration, and tumor-related angiogenesis [126, 150, 190]. K_Ca_3.1 interacts functionally with K_v_1.3 and CRAC [126, 190].

### 8.4. K_Ca_2.2

K_Ca_2.2 (SKCa2), small conductance K_Ca_ channel, is expressed in a variety of healthy tissues, including brain and muscle [191]. It was also described in Jurkat leukemic cells, where its functional expression channels were high, comparable to the K_Ca_3.1 expression in activated healthy T cells [189, 192, 193]. K_Ca_2.2 is activated by Ca^2+^ with an apparent K_d_ = 700 nM and is upregulated by p56Lck [192]. It has a higher expression in G_0_/G_1_ compared to G2/M phase, supporting a sustained Ca^2+^ influx [194]. In Jurkat, K_Ca_2.2 silencing but not K_v_1.3 inhibition suppresses the Ca^2+^ entry; it can be restored by the ectopic expression of K_Ca_3.1 [193]. This study obviously argues that K_Ca_2.2 and K_Ca_3.1 may be interchangeable, but it is not clear to which extent. It should be noted that in Jurkat the functional expression of K_Ca_2.2 channels is constitutively high and can be compared to the K_Ca_3.1 expression in activated healthy T cells [189, 193]. Remarkably, mitogenic stimulation instead of increase causes a 2-3-fold decrease of the K_Ca_2.2 expression both at mRNA and functional expression in the membrane levels [192].

### 8.5. Tandem-Pore Domain (K2P) Channels

K2P represents K^+^ selective channels, genetically unrelated to the former two families (K_Ca_ and K_V_). Each channel subunit possesses two pore domains, and a minimal number of transmembrane segments, two per pore, as compared to the 6-TM subunit structure of K_Ca_ and K_V_. Consequently, functional K2P channels are dimers; functional heterodimers were reported only for closely related TASK1 and TASK3, and the rest of active K2P channels are homodimers. There are 14 members of K2P family discovered in mammals (see [135] for a review). The activity of K2P channels is practically independent of the membrane voltage; rather, they are regulated by a variety of metabolic and physical factors. First reports on the functional expression of TASK1 and 3 in T cells [195] and of TRESK in Jurkat leukemic T cells [196, 197] in human models came relatively recently. Further on, TASK2 was found to be constitutively expressed in human T cells, but not in B or NK cells. Within T cells the relative expression was dependent on the T cells subset; TASK2 was strongly upregulated in CD4^+^ and CD8^+^ cells of patients with multiple sclerosis [198]. Later on it was shown that TASK2 expression in CD4^+^ T cells strongly correlates with rheumatoid arthritis disease activity [199]. Mechanistic explications of both correlations are still lacking. However, roles of TASK1 and TASK3 in T cells function seem to be clearer. TASK1 and 3 belong to acid-sensitive K2P, which are inhibited by low external pH; for TASK1 and TASK1/3 heterodimer apparent pK is 7.3 and TASK3 homodimer displays pH-sensitivity outside the physiological range; intracellular pH changes were inefficient for both channels [200]. TASK channels are directly inhibited by anandamide and some synthetic cannabinoides. They are also inhibited by the stimulation of G_q_-coupled receptors, possibly via the breakdown of PIP_2_, with TASK1 and TASK1/3 heterodimer being more sensitive than TASK3 homodimer [135]. According to the data of Meuth and coworkers (2008) TASK channels may account for up to 40% of the outward K^+^ current in human T cells; their inhibition caused a significant decrease in production of cytokines and cells proliferation [195]. In addition, the role of TASK2 and TRESK in the RVD is comparable to that of K_v_1.3, whereas TASK-3 shows a lower contribution, which was somewhat higher in activated T cells [145]. TASK-3 is often amplified in different types of cancer; it is also expressed in mitochondrial inner membrane, which likely underlies its role in apoptosis. Yet its impact on the oncogenesis may be not necessarily negative and in some cases its high expression correlates with a better prognosis [128]. No studies of TASK expression in T-ALL are available to date. With TRESK the situation is different. Normally, it not only is abundantly expressed in neurons of dorsal ganglia, but also is reported in spleen and thymus in murine models [201]. Yet TRESK is strongly upregulated in several leukemic cell lines as well as in patients with T-ALL [197].* In vivo* real time K^+^ flux measurements and concurrent patch-clamp data on Jurkat cells revealed that both TRESK and K_v_1.3 mediate AVD in the intrinsic apoptosis pathway, yet TRESK is transiently upregulated by apoptotic stimulus (staurosporine) and then completely inactivated in a half of hour, causing a strong membrane depolarization, whereas the K_v_1.3 contribution to the K^+^ efflux was more constant in time [147]. TRESK, which does not have close homologues, is unique among K2P channels, because its principle way of activation is Ca^2+^-dependent (Figure 3(c)). TRESK possesses a large cytosolic loop between transmembrane segments 2 and 3, which harbors, similar to NFAT, the Cn docking site (the NFAT-like domain) and several phosphorylation sites. Phosphorylation by MARK and PKA in the two distinct sites caused TRESK inhibition. On the contrary, binding of Ca^2+^-calcineurin to NFAT-like domain and dephosphorylation at both sites provoked the TRESK activation [202]. This is a very important point, as activation of the NFAT-pathway by calcineurin at prolonged Ca^2+^ increase would cause also the TRESK activation. TRESK activation may be not only the consequence of a prolonged Ca^2+^ increase, but also its cause, due to a feedback support of the Ca^2+^ influx via the TRESK-mediated membrane hyperpolarization (Figure 3(c)). TRESK dependence on Ca^2+^ is different from that of K_Ca_ channels. K_Ca_ are directly activated by Ca^2+^ increase and immediately deactivated after Ca^2+^ removal. TRESK in contrast, once activated via dephosphorylation by calcineurin, could maintain its activity for a longer period after the Ca^2+^ removal. Thus, TRESK could be directly involved in the gene expression in T-ALL and may be considered as a plausible target for the immunomodulation [203, 204]. Yet, it is a general problem with K2P channels, and there are no specific blockers of any of mammal K2P channels, which handicaps studies of their roles* in vivo* as well as K2P-targetted therapies. However, recent advances in studies of low-molecular weight modulators for K2P are promising. In particular, antihistamine loratadine appears to block TRESK with a high affinity and does not display drastic collateral effects [205].

### 8.6. SOCE/CRAC (Orai1-STIM1)

**Figure 4:**
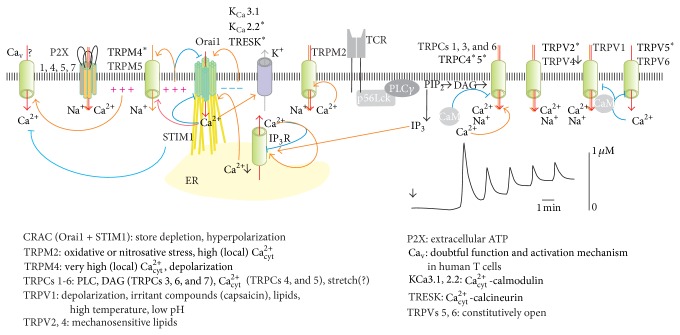
Ca^2+^ influx network in T cells. Channels, marked with asterisks, are overexpressed or present exclusively in T-ALL. Central is activation of CRAC- (Orai1+STIM1) mediated Ca^2+^ influx. Activation of PLC (e.g., via the T cell receptor) causes cleavage of PIP_2_ with the production of DAG and soluble IP_3_; the latter activates IP_3_-receptor Ca^2+^ release channels of the endoplasmic reticulum. Ca^2+^ store depletion is sensed by specialized transmembrane sensors (STIM1), which oligomerize and move to special contact zones of ER with the plasma membrane, where they physically interact with the channel-forming proteins (Orai), forcing them to form active Ca^2+^ selective channel (CRAC), which mediates Ca^2+^ influx. Operation of CRAC is further modulated by the activity of other channels, which affects the membrane polarization and intracellular Ca^2+^. Voltage-independent Ca^2+^-dependent K^+^ channels potentiate CRAC-mediated Ca^2+^ influx, lowering the membrane potential, thus increasing the driving force for Ca^2+^ uptake. Conversely, channels with a predominant Na^+^ permeability (TRPM4) cause membrane depolarization and abrogation of the Ca^2+^ influx. Depending on the channel selectivity high Ca^2+^ (e.g., TRPV5) or indiscriminate Ca^2+^/Na^+^ (e.g., TRPC) as well as (when applicable) on the nature of the feedback (positive or negative, see respective loops) via Ca^2+^ and on the context (differential ways for the activation of particular ion channel), overall Ca^2+^ signal can be positively or negatively modulated. An idealized Ca^2+^ response to a mitogen stimulation, which contains both oscillatory and monotonous increase components, evidencing a feedback regulation via Ca^2+^, is given as an example. Ways of the channels' activation are summarized below. From the left to the right: Ca_v_ (voltage-dependent Ca^2+^ channels), P2X (purinergic ionotropic receptors), TRP (transient receptor potential channels), Orai-STIM1 (CRAC, Ca^2+^ release-activated Ca^2+^ channel), K_Ca_ (Ca^2+^-activated K^+^ channels), TRESK (TWIK-like spinal cord K^+^ channel), and CaM (calmodulin).

Ca^2+^ signaling in T cells differs greatly from those in excitable cells, which mainly rely on the voltage-dependent (depolarization-activated) Ca^2+^ channels of the plasma membrane. In lymphocytes, contrary to this, Ca^2+^ rise in cytosol is mediated by the store-operated Ca^2+^ entry (SOCE), named also CRAC, for Ca^2+^ release-activated Ca^2+^ current, that is, Ca^2+^-selective current of the plasma membrane, activated by the Ca^2+^ depletion in the ER. It is extremely (factor > 1000) selective for Ca^2+^ over monovalent cations and has extremely low single channel conductance for Ca^2+^ (30 fS), which is compensated by a very high channels density (ca. 100 channels/*μ*m^2^). Due to its intrinsic inward rectification, Ca^2+^ influx via CRAC is strongly potentiated by membrane hyperpolarization, whereas depolarization reduces Ca^2+^ entry in lymphocytes [126]. For a long time it was thought that CRAC is mediated by members of TRPC subfamily (see below). Of course, these relatively weakly selective channels alone may not be responsible for a strongly Ca^2+^-selective CRAC, but now there is also an ample evidence that TRPCs and CRAC are functionally and physically separated [206]. Crucial for molecular identification of CRAC was a study of severe combined immune deficiency (SCID), which was characterized by nonfunctional CRAC in T cells from some patients. In such a way, Orai1 was discovered as a pore-forming protein of CRAC, as single mutation in Orai1 from SCID patients was responsible for a defective CRAC function [207]. Orai does not relate to any known ion channel. In humans, three different isoforms form very similar CRAC channels, but in lymphocytes only Orai1 seems to be of functional importance [208]. Store depletion is communicated to Orai via STIM (stromal interaction molecule) proteins, located in the ER-membrane. In Ca^2+^ replete stores STIM proteins are randomly distributed at the membrane surface, and store depletion causes oligomerization of STIM in special contact areas with plasma membrane, where cytosolically exposed STIM domain directly interacted with both N- and C termini of Orai1, thus, causing CRAC activation (Figure 4, see also [208] for a recent review). There are two STIM isoforms in T cells, and both are important for CRAC, yet in murine models STIM1 or STIM2 deficiency caused a complete or partial abolishment of CRAC, respectively [209]. CRAC plays a central role in cytokines production, firstly, via Ca^2+^ activation of the NFAT transcription factor; conversely, it does not play a very significant role in the antibodies production by B cells (see [208] and references therein). Orai1 displays Orai1 displays two-time lower current density in Jurkat lymphoblasts as compared to resting T-cells; no significant difference in STIM1 expression was revealed between these two cellular models [210]. Relatively modest changes in the CRAC expression per se may not underlie changes in Ca^2+^ signaling in activated and malignant T cells. More likely, differences in the expression and regulation of “partner” K^+^ channels, especially those activated by intracellular Ca^2+^ rise, may be more important for the modulation of the CRAC function (Figure 4). As CRAC-mediated Ca^2+^-influx is inhibited by inflowing Ca^2+^ [211] and membrane depolarization, its activity may be further modulated by TRPs. TRPs differ greatly not only in the modes of their activation and expression in leukemic T-cells (see below), but also by their Ca^2+^/Na^+^ selectivity, hence differentially affecting membrane depolarization and Ca^2+^ signal. Figure 4 represents possible cross-talks between plasma membrane cation channels, including a feedback, provided by their differential dependence on the cytosolic Ca^2+^. More scenarios, exploiting TRP and ORAI competition for STIM1, physical interactions, affecting ORAI surface expression and membrane localization, or existence of hybrid SOCE channels are discussed in the recent review by Saul and coworkers (2014) [212].

### 8.7. TRP (Transient Receptor Potential) Family Channels

TRP channels (currently, 28 described in mammals) are cationic, mostly nonselective and Ca^2+^-permeable ones; they resemble by transmembrane topology but distantly are related to voltage-gated (e.g., K_v_) channels [213, 214]. They are subdivided into TRPC (canonical), TRPM (melastatin), TRPV (vanilloid), TRPA (ankyrin), TRPML (mucolipin), and TRPP (polycystin) subfamilies. TRPA, TRPP, and TRPML detection in lymphocytes was not addressed. However, TRPA may have a relatively restricted expression, mainly in sensory neurons, whereas TRPML forms intracellular channels [213].

TRPC 1, 3, and 6 were reported in PBL and Jurkat cells, Jurkat expresses additionally TRPC 4 and 5 [124]. All above channels are weakly selective (P_Ca_/P_Na_ = 1–5), with linear or dual-rectifying I/V relation [215]. TRPC channels at cytosolic C-terminus contain two specific Ca^2+^-binding sites, a EF-hand and CIRB, a calmodulin/IP3R-binding domain and may be directly regulated by intracellular Ca^2+^ [213]. Mostly, they are inhibited via Ca^2+^/CaM pathway [216]. According to their properties, TRPC can be subdivided into TRPC1/4/5 and TRPC3/6/7 (*trpc2* is a pseudogene in humans) subgroups. A common property of the latter subgroup is their activation by DAG (or its membrane permeable form OAG), whereas TRPC1/4/5, albeit being dependent on the PLC activity, are not responding to DAG [217]. OAG-activated current mediated the influx of Ca^2+^, Ba^2+^, and Sr^2+^ into Jurkat and PBLs; on the contrary, in case of SOCE (CRAC) activation cation influx was Ca^2+^-selective, without measurable Ba^2+^ component [218]. In HPB-ALL, acute T lymphoid leukemia only TRPC1 was expressed at detectable levels among TRPCs 1 to 7, as compared to Jurkat, expressing TRPCs 1, 3, and 6 and more (see below). Ca^2+^ influx, induced in HPB-ALL cells by Δ^9^-tetrahydrocannabinol, was less sensitive to SOCE and, based on pharmacological evidence, was mediated by TRPC1 via CBD-2 receptors and DAG [219]. Yet there are some doubts that TRPC1 could form functional homomeric channels, whereas it definitely can form heteromeric channels with all others TRPCs, including 4 and 5 [220] as well as with TRPV4 [221]; in the latter case translocation of TRPC1/TRPV4 complex to plasma membrane requires store depletion and STIM1 involvement. TRPCs 4 and 5, which are not present in PBLs, are robustly expressed in Jurkat cells [124]. Importantly, homomeric TRPC 5 or heteromeric TRPCs 4 and 5 act as a positive loop for the NO production: channel's nitrosylation provokes a formation of disulphide bond between neighboring cysteine residues, locking the channel in the open conformation, thus, promoting Ca^2+^ influx and Ca^2+^-dependent NO production [222]. It remains to be elucidated whether such mechanism may operate in leukemic T cells. Partial silencing of TRPC3 in CD4^+^ caused a reduction of intracellular Ca^2+^ only under limiting external Ca^2+^ (<0.1 mM) and slightly (<20%) decreased the proliferation of activated cells [124]. However, specific TRPC3 blocker, Pyr-3, strongly reduced the late phase of Ca^2+^ entry induced by anti-CD3 antibodies in Jurkat cells [223], confirming the TRPC3 role in Ca^2+^ signaling.

TRPM2 form nonrectifying channels, almost equally permeable to K^+^, Na^+^, and Ca^2+^ [224] and slightly less to Mg^2+^ [225]. TRPM2 is known as “chanzyme” because its dual function of ion channel and C-terminal enzyme domain [226]. (TRPM2) mRNA was highly abundant in CD34^+^ precursors and in hematologic malignant cell lines of different lineages, like Jurkat cells (T-ALL), K562 (erythromyeloblastoid leukemia), AML-193 (acute monocytic leukemia), U937-ecoR (monocytic leukemia), and TF-1 (erythroleukemia) [224, 227, 228]. TRPM2 is activated by reactive oxygen species, and this way may be the principle route of TRPM2 activation in Jurkat cells, whereas intracellular ligands, like cADPR, appear to be inefficient for this model system [229]. The combination of channels responsible for Ca^2+^ influx varied depending on the method of stimulation. In Jurkat cells, activation by ConA led to late Ca^2+^ influx via TRPM2 without CRAC involvement, whereas TRPC (TRPC3?) channels were responsible for anti-CD3-activated Ca^2+^ influx [223]. In CD4^+^ T cells of healthy donors, stimulation by anti-CD3/anti-CD28, caused a transient increase of the TRPM2 expression [124]. TRPM2 mediates apoptosis upon the oxidative stress [227]. Overexpression of TRPM2 increases tumor susceptibility to oxidative stress, favoring the mitochondrial Ca^2+^ overload and triggering the intrinsic pathway of apoptosis, so TRPM2 is considered as a tumor suppressing factor [230].

TRPM4 encodes an intrinsically voltage-dependent outwardly-rectifying [231] monovalent cation channel, equally permeable to Na^+^ and K^+^ [232]. It requires high (micromolar) cytosolic Ca^2+^ for its activation [231]. Wenning et al. (2011) reported nondetectable levels of TRPM4 mRNA in naïve T cells, but a consistent expression both in Jurkat and effector CD4^+^ cells; TRPM5 was expressed in stimulated and nonstimulated PBLs and Jurkat. TRPM4 and TRPM5 possess similar biophysical characteristics and are unique among TRP superfamily, which are virtually Ca^2+^ impermeable [124]. Thus, under physiological conditions TRPM4 mediates Na^+^ influx, producing membrane depolarization [232]. This tends to reduce the CRAC-mediated Ca^2+^ influx. Indeed, suppression of TRPM4 in Jurkat transforms PHA-induced Ca^2+^ signal from oscillations to a higher sustained Ca^2+^ increase, resulting in a higher IL-2 production [232]. Similarly to TRPM4, TRPM5 is activated by high intracellular Ca^2+^, but, in contrast to the former, it is strongly suppressed by external acidification [233], which may be relevant physiological situation under hypoxia in lesions. Analysis of polymorphisms in TRPM5 genes revealed correlation of childhood leukemia with certain genotypes [234]. Interestingly, TRPM5 gene is located in the human chromosome 11, which frequently shows aberrations for a number of hematological malignancies, including ALL [235]. In addition, on the protein level TRPM5 is conservatively regulated by residual proteins, formed in the Notch pathway, which in turn is important pathway associated with T-ALL [236]. However, in contrast to TRPM4, direct experimental demonstration of the TRPM5 channels activity in T cells is lacking so far.

TRPM7 is another, in addition to TRPM2, “chanzyme,” consisting of ionotropic divalent cation-permeable channel and kinase, linked to cytosolic C-terminus [237]. It is ubiquitously expressed, but normally at relatively low levels [213].* In vivo* current, generated by TRPM7 channels, is strongly outwardly rectifying, due to a blockage by cytosolic Mg^2+^ and, possibly, some additional internal factor [213]. Mg^2+^ block of TRPM7 channel and a role of a very similar TRPM6 channel in Mg^2+^ homeostasis led to a hypothesis that TRPM7 could equally participate in sensing and control of the intracellular Mg^2+^. Yet, current experimental evidence is more against that in favor of this hypothesis [208, 213]. TRPM7 expression is higher in Jurkat as compared to healthy T cells [124]. Importantly, cell-specific TRPM7 deletion arrests T cells differentiation at the DN stage [89]. Although TRPM7 is the only channel up to date with an approved role in the lymphocyte development, it is not known, whether this role is mediated by Mg^2+^ or Ca^2+^ influx through its pore, kinase domain activity, or a combination of these factors. Interestingly, TRPM8, a crucial motor element for cell migration in glioblastomas [238] but oppositely acting in other tumor types (see [230] for a review), is expressed neither in healthy PBLs nor in Jurkat lymphoblasts [124].

There are two subgroups in the TRPV family: TRPV1/4 and highly Ca^2+^-selective TRPV5/6 ones. TRPV1 (vanilloid receptor), the founding member, represents an outward-rectifying voltage-gated channel, which is upregulated by different physical factors (external pH, heat) and a great variety of unrelated chemical compounds (Figure 4) and is modulated by protein phosphorylation by PKA and PKC [239]. Its pore is flexible and the channel selectivity changes upon the activation and it depends also on the activation factor [216, 240]. TRPV1 is modestly selective for Ca^2+^ over Na^+^, given values between 10 for capsaicin and 4 for heat [241]. Thus, the fraction of Ca^2+^ current inflowing the cell via TRPV1 counts only a few percent of the total current, mainly carried by Na^+^. Yet, this Ca^2+^ influx is sufficient to cause the TRPV1 desensitization, which likely is caused by Cn-mediated dephosphorylation of several sites [216]. TRPV1/4 can be also considered as chemosensors; among the agonists for TRPV1, 2 and 4, cannabinoids should be mentioned [216, 242].

TRPV1 and TRPV2 are the most expressed among TRPV1/4 channels in human peripheral blood and leukocytes, with the TRPV2 mRNA being more abundant than that of TRPV1 by a factor of 150 and 20, respectively [243, 244]. TRPV2 is relatively poor expressed in other tissues (except smooth muscle and lung) but in blood [245]. TRPV2 is expressed in lymphoma and leukemic cell lines, including T-ALL, Jurkat, and MOLT-4 [124, 245, 246]. On note, TRPV2 activation by cannabidiol in some tumors increased their sensitivity to anticancer drugs, by increasing drugs uptake and stimulating cell death [235, 247]. TRPV2 was also detected in the intracellular (endosomal) membrane, where it mediates Ca^2+^ release [248]. There are two splicing variants of TRPV2: full-length and poreless short (s-TRPV2) ones. S-TRPV2 is more typical for tumor cells including leukemic ones; it is localized in cytosol, and it inhibits full TRPV2 translocation to the cell membrane [246]. TRPV2 is probably one of the least understood TRP channels when it comes to its regulation. It has a bipolar current-voltage dependence and similar permeability for Ca^2+^ and Na^+^ [249]. In humans, in contrast to rats, TRPV2 appears to be not activated by noxious heat [250]. It is thought that the main way of its activity regulation is the mobilization from the ER to the plasma membrane, for example, by growth factors [246, 251]. However, in murine aortic myocytes and neurons, TRPV2 is activated by membrane stretch and mediated a Ca^2+^ influx [252, 253]. Activation by mechanical stretch and hypotonicity was demonstrated also for TRPV2-like channels, expressed in human mast-cell line [254]. Our own study on Jurkat cells revealed that the properties of major mechanosensitive channel in their plasma membrane are indistinguishable from TRPV2 by its voltage dependence, cation selectivity, and pharmacological profile (Pottosin et al., unpublished). Properties of mechanosensitive currents in Jurkat were clearly distinct from TRPV1 and 3 but may be confused with those generated by TRPV4 [255], albeit the latter channel shows a somewhat higher Ca^2+^ over Na^+^ selectivity. TRPV4 channel is also proved to be mechano- and osmosensing element in different tissues [256–258]. It is regulated by intracellular Ca^2+^ in a complex way, via CaM binding to C- and N-termini; it activates at moderate intracellular Ca^2+^ increase and inactivates at higher (>800 nM) Ca^2+^ levels [256]. It is still a matter of debate, whether this activation may be direct or mediated by mechanosensitive phospholipase A2 activity, which metabolizes the arachidonic acid and produced epoxyeicosatrienoic acid, which in turn activates the TRPV4 [213]. We give lesser preference to TRPV4 versus TRPV2 due to their very low expression in Jurkat cells [124], but this point requires further exploration. Emerging evidence is accumulated that TRPV2 could colocalize within a network of K_v_1.3, K_Ca_3.1, and CRAC, thus contributing to a variety of vital T cells functions by modulation of the Ca^2+^ signaling [245]. In Jurkat cells and mouse thymocytes, the RVD in response to hypotonic treatment is a Ca^2+^-dependent process, unlike mature peripheral lymphocytes, either mouse or human, where hypotonic stress does not provoke any intracellular Ca^2+^ change [150]. About respective Ca^2+^ signal it is known that it is mediated by the plasma membrane nonselective channels (TRPs?), which are 100-time less sensitive to Gd^3+^ as compared to the CRAC. Likewise, in immature T cells and T-ALL the volume regulation is controlled by a mechanosensitive TRP, which, in accordance with our data, could be the TRPV2.

TRPV5 and 6 are inwardly rectifying and the only highly Ca^2+^-selective (P_Ca_/P_Na_ > 100) members of the TRP family [213, 215]. Both TRPV5 and TRPV6 channels could be found in resting human PBL and Jurkat cells, as transcripts and, functionally, based on the different sensitivity of measured single-channel currents to ruthenium red (RR). However, TRPV6 expression in Jurkat cells and PHA-activated PBLs is much higher as compared to resting cells, implying a stimulating role of TRPV in the proliferation. Indeed, inhibition of TRPV currents by RR arrested the progression of the cell cycle in activated PBLs or Jurkat in G0/G1 and S or G2/M phases, respectively [259]. TRPV6 in Jurkat may physically interact with or even contribute to the CRAC/SOCE [260].

### 8.8. Purinoreceptors (P2X)

P2X forms nonselective Ca^2+^-permeable channels, activated by external ATP [261]. First evidence that peripheral blood T cells bear P2X, whose activation by ATP produces depolarization and Ca^2+^ influx, was obtained by Baricordi and coworkers (1996). It was demonstrated that P2X may contribute to T cell proliferation, induced by mitogenic stimulation [262]. There are seven P2X subtypes (P2X1-7), of which mainly P2X1, P2X4, and P2X7 were expressed in primary CD4^+^ and Jurkat cells [263, 264]. T cell activation was shown to induce ATP release and considerable augment of P2X1, P2X4 [264], and P2X7 [263] expression. In turn, stimulation by ATP contributes to Ca^2+^ rise and enhances IL-2 production [263, 264]. Silencing or chemical inhibition of P2X receptors strongly impair Ca^2+^ influx, NFAT activation, and interleukin production [263, 264]. Therefore P2X along with STIM1 and Orai1 was suggested to contribute to Ca^2+^ entry, providing an amplification mechanism for TCR signaling [263, 264]. ATP release required for P2X stimulation may be achieved by exocytosis, or from dying cells, but also from intact T cells [265] via some wide-pore channels, like pannexin 1 [266] or, notably, through P2X7 themselves. Importantly, stimulation of T cells or Jurkat causes a rapid (within minutes) translocation and clustering of P2X1 and P2X4, but there are no changes in the P2X7 distribution [264]. The same is true for the immunological synapse, where in addition pannexin 1 is rapidly recruited. It is hypothesized that such a colocalization may produce a strong positive purinergic feedback, with a further amplification due to the concentration of ATP in the synaptic cleft, thus, allowing sensation of just few presented antigens [261]. Additionally, P2X7 seems to interact with apoptotic pathways: prolonged activation of this receptor by extracellular ATP released by neighboring apoptotic cells promotes overall apoptotic process [267].

Remarkably, the evidences emerged, which link P2X7 with leukemogenesis. Bone marrow samples obtained from patients with different types of leukemias, mainly AML and CLL, show a substantially higher level of P2X7 mRNA expression as compared to normal donor group [268]. Quite a few samples from ALL patients were analyzed in this study, and, although they showed increased level of P2X7, it was not indicated if there were B-ALL or T-ALL cells. In contrast, recent study on identification of interconnected markers for T-ALL, which included 173 T-ALL patients, revealed P2X7 as non-DE gene [125]. As was mentioned earlier, non-DE genes may be involved in signaling networks, upregulated during leukemogenesis.

### 8.9. Cl^−^ Currents

Any osmotic or volume adjustment (e.g., RVD or AVD) requires a massive transport of solute across the plasma membrane. Such a massive transport is possible only when it occurs in electroneutral manner, so that cations (e.g., K^+^) transport needs to be accompanied by a parallel transport of anions via anion channels. In ALL (T-ALL) the expression of ClC2-5 was detected by PCR; ClC-3 is robustly expressed in healthy PBL and leukemias, whereas ClC-2 is mainly in leukemias [269]. ClC2 and ClC3 play roles in volume regulation, whereas there are ClC2 and ClC4- in pH homeostasis. Activator-induced proliferation of healthy T cells involved DIDS-sensitive NPPB-insensitive Cl^−^ channels, whereas in case of leukemic cells (Daudi, Jurkat, and H-60) it is mediated by DIDS-insensitive NPPB-sensitive ones (but not ClC-2, as evidenced by silencing experiment) [269, 270]. VSOR (Cl_swell_) (volume sensitive outward rectifying Cl^−^ current, activated by swelling) was first discovered in peripheric human T cells [271–273]. Identical biophysical properties (intrinsically outward-rectifying) Cl^−^ current for CFTR (cystic fibrosis transmembrane conductance regulator) cAMP-activated [274] and volume-regulated [273] currents were reported also for leukemic Jurkat cells. VSOR may also require lck-kinase (p56^lck^) activity: the inhibition of this kinase blocks swelling-induced Cl^−^ current, whereas addition of p56^lck^ to excised patches caused the current activation [275]. Once activated, VSOR may stay extremely long time in open state (several minutes) without transitions to closed state. Yet more careful inspection revealed close-open events. At physiological ionic conditions and zero membrane voltage unitary conductance of VSOR is about 40 pS [274–276]; single channel current displays a strong outward rectification, which mimics the voltage dependence of the whole cell VSOR. Likewise, VSOR is encoded by CFTR, unique member of ABC-transporter family, encoding Cl^−^ channel and not a pump. Transfection of the CFTR-defective B cells and lymphoblasts with wild-type CFTR cDNA resulted in a restoration of a cell-cycle-dependent (maximal expression in the G1 phase, low expression in the S-phase) cAMP—activated outward rectifying Cl^−^ current [277, 278]. It was shown that the outward rectifying Cl^−^ current can be activated in a triple manner: transiently by cAMP and steadily by either hypotonic stress or intracellular Ca^2+^ increase; only the activation by cAMP was abolished by genetic defect in CFTR [279]. In addition, CFTR deficient mice have shown a higher NFAT to nucleus translocation and higher interleukin and immunoglobulin E production by T cells due to altered (elevated) intracellular Ca^2+^ increase in response to T cell receptor activation. This may be a depolarization, caused by outward-rectifying Cl^−^ current which normally moderates Ca^2+^ influx via CRAC [280]. Summarizing, all aforementioned Cl^−^ channels are expressed in normal lymphocytes and lymphoblasts. Whereas in case of ClC channels direct demonstration of respective currents is lacking, in case of VSOR its molecular identity remains to be revealed. Preliminary pharmacological analysis suggests differential dominant Cl^−^ currents in normal and malignant lymphocytes.

### 8.10. Other Channels

Nicotinic AchR forms nonselective cation channel, with important roles in neoplastic progression, both via conducting and nonconducting signaling mechanisms. There is circumvent evidence for nAchR expression in lymphocytes and nicotine affects hematopoiesis [281, 282]. Yet functional roles of nAchR and cholinergic signaling in healthy lymphocytes and leukemias remain to be elucidated. Both human T and leukemic Jurkat cells appear to express ionotropic glutamate receptors of AMPA and NMDA types. Their activation by respective ligands promoted cell adhesion and migration [283, 284]. NMDA receptor antagonists decreased proliferation of T and Jurkat cells, but in a different manner. In Jurkat cells this reduction was associated with G1-S transition arrest and increased apoptosis; neither phenomenon was associated with a decrease of proliferation in healthy T cells [284, 285]. In early studies one may find the notion that a few percent of T cells from human peripheric blood express functional voltage-dependent Na^+^ channels [159], but their role in nonexcitable cells remains obscure. Expression of *α*-subunits of voltage-dependent L-type Ca^2+^ channels (Ca_v_1) as well as interacting *β*-subunits is consistently reported in human healthy T and Jurkat cells. However, up to now neither depolarization-activated Ca^2+^ selective currents nor significant Ca^2+^ influx evoked by a depolarization was detected in human T cells [126, 208]. However, on murine models, typical L-type Ca^2+^ currents, which disappeared upon Ca_v_1.4 silencing, were reported in a single study [286]. Furthermore, mice with mutant *β*-subunits or Cav1.4-deficient mice, or mice with knock-down Ca_v_1.2 and/or Ca_v_1.3 display clear immunopathological phenotypes (see [208, 287] for a recent update). Yet no immunopathological phenotypes were demonstrated in humans. The situation may be even more complicated due to the fact that STIM1, activating CRAC, inhibits the L-type Ca^2+^ channel activity [288], so that CRAC dominance in T cells implies a suppression of the Ca_V_ activity. In the latter study, authors were able to show a depolarization-activated Ca^2+^ increase in mutant STIM1-deficient Jurkat cells, transfected with Ca_v_1.2 along with *β*1b and *α*2*δ*1 subunits; yet without such a transfection no significant depolarization-activated Ca^2+^ influx was detected, which questions again the functional role of background Ca_v_1 channels. It was speculated that instead of activation by large depolarization, hardly attainable in T-cells, Ca_v_ channels may be activated due to their clustering and interaction with PKC [287].

## 9. Targeting Ion Channels for the T-ALL Treatment

Although an impressive progress has been made in the treatment of T-ALL, it is still a disease with enormous need for innovation in the therapeutic field. Undoubtedly, the concept of total chemotherapy has been a milestone in the history of the treatment of this disease. The stage of differentiation, at which the proliferative arrest occurs, denotes not only the degree of maturation of the leukemic cell, but also its clinical behavior and response to a particular treatment. Early T leukemias, regarding the mature T cell neoplasias, are more chemoresistant and, therefore, demonstrate lower rates of complete remission and overall survival. The current therapeutic strategy is based on a combination of chemotherapeutic agents targeting DNA and protein synthesis (methotrexate, L-asparaginase, doxorubicin, cyclophosphamide, cytarabine, and nelarabine), or inducing apoptosis (dexamethasone). Allogeneic bone marrow transplantation has been shown to be beneficial in a selected population in this leukemia subtype. Although the leukemia-free survival has significantly improved in the last decades, being reported in some series in children up to 85% at 5 years [289], traditional antileukemic drugs demonstrate numerous short- and long-term toxic side effects, which may lead to significant morbidity. More than 50% of pediatric cancer survivors will develop long-term complications, including cardiovascular, gonadal, and gastrointestinal/hepatic dysfunction, neurocognitive sequelae, auditory complications, and decline in growth, with approximately 25% of cases being severe [290]. Additionally, long-term toxicities of these antineoplastics include the possibility of a future secondary malignancy.

Among novel molecular targets FLT3, JAK1/JAK3 and Notch were proposed [291]. Some FLT3 inhibitors as lestaurtinib currently have been studied in the phase III clinical trials, showing* in vitro* apoptosis induction capability in LLA cells lines that express high levels of FLT3 and interacting synergistically with multiple chemotherapeutic agents. JAK inhibitors are currently in varying stages of development and they have only been in adult trials. Notch inhibitors are under development. Though, in animal models and in the phase I clinical trials, serious gastrointestinal toxicity has been shown, which can be reversed by concomitant use of glucocorticoids, subsequent additions have been few and mainly restricted to the advent of some new chemotherapeutic drugs (clofarabine, nelarabine), the use of monoclonal antibodies such as alemtuzumab, and more recently drugs able to impact molecular targets (see for review [292]). Sustained activation of Cn/NFAT pathway is a hallmark of the T-ALL [43, 107, 108]. However, this pathway is ubiquitous not only in malignant, but in many healthy tissues as well. As a result, although administration of the Cn inhibitors showed an antileukemic effect [43, 77], it was associated with a number of undesirable offtarget effects [293].

Leukemias show altered expression of a variety of ion channels (for review see [127] and this review). Even if the profile of channel expression is not changed, as was shown for nondifferentially expressed genes, it may be involved in newly upregulated signaling network [125]. However it is unlikely that these alterations by themselves launch the leukemic scenario. Rather, ion channels represent important components, which ensure homeostatic conditions favorable for the migration, proliferation, and leukemic survival, or facilitate the expression of oncogenes, involved in the leukemogenesis. Almost all signaling pathways, upregulated in the T-ALL, show a dependence on the Ca^2+^ signaling. Channels may be involved in the cell motility and tumor metastasis. These phenomena may provide the basis for the targeting ion channel in leukemogenesis.

For example, TRESK channels are abnormally expressed in some types of T leukemias [197]. As was mentioned, this channel is activated through Cn-dependent mechanism and is involved in a positive feedback regulation of the Ca^2+^ entry. Thus, TRESK inhibition could result in a downregulation of the Cn. However, the issue is much more complex than it might seem. First of all, TRESK is normally expressed in some healthy tissues [201], foremost in the central nervous system where it is related to the migraine pathology [294]. Next, no specific blockers for the TRESK are known so far. To resolve a similar problem related to the K_v_10.1 channel, specific functional antibodies were designed [295]. Although it is possible that an antibody actively crosses the blood-brain barrier, the concentrations efficient for tumors are likely lower than those required to reach the brain parenchyma and to cause massive side effects [295]. Another classical example is K_v_11.1, aberrantly expressed in many tumor types [127]. The difficulty lies in the fact that K_v_11.1 normal localization is the cardiac tissue, where it contributes in the repolarization phase of the cardiac action potential [175]. Although specific blockers of this channel are available, its inhibition can retard the repolarization and prolong QT interval leading to the ventricular arrhythmia, with a possible fatal fibrillation [296]. Therefore, K_v_11.1 is generally considered as an undesired pharmacological target. To overcome this issue, it was proposed to consider different blocking mechanisms and target a particular conformational state of leukemic ion channels, as well as bipolar antibodies, raised against the complex of the channel and one of its auxiliary proteins, characteristic for tumors [127].

Yet another very important point should be taken into consideration, namely, hierarchical tumor development, its genetic instability, and heterogeneity of the T-ALL population. Obviously, LSC and T-ALL clones, representing the same clinical case, differ in their “gene expression signatures” and, accordingly, in their sensitivity/resistance to chemotherapy. Chemotherapeutic agents, used nowadays, successfully eradicate the blast cells in many patients; however, they have very little if any effect at the level of the blast progenitor cell, namely, LSC, which is biologically distinct from most of the cells found in a typical patient [297]. Then LSCs that survived chemotherapeutic attack finally cause the chemoresistance and relapse. Additionally, some therapeutic targets may be present only in a few nondominant clones, which does cause unresponsiveness to the treatment. Targeting the LSC was suggested as the Holy Grail of leukemia therapy [298]. Then, considering ion channels as a possible target, the search for ion channels expressed specifically in LSC would be of a special advantage. We suggest that the experiments with primary blast cells or cell lines derived from patients in relapse may give some important information.

## 10. Conclusions

Tumor-specific expression of a certain ion channel is a relatively rare phenomenon. However, changes in the expression (including its variation during cell cycle) and subcellular localization, splicing of channels (and relative expression of different variants), role of heteroligomerization, of *β*-subunits, and of other auxiliary proteins (especially integrins), and modification of the channel protein, as well as every aspect of microenvironment and metabolic regulation and signaling pathways context, affecting the channel's activity, may be of potential importance for the tumor progression. Here, the integration of the channels function and their crosstalks, most apparently via cell membrane polarization and intracellular Ca^2+^ changes, needs to be considered.
